# Cytotoxic properties, glycolytic effects and high-resolution respirometry mitochondrial activities of *Eriocephalus racemosus* against MDA-MB 231 triple-negative breast cancer

**DOI:** 10.1186/s12906-024-04615-x

**Published:** 2024-09-10

**Authors:** Francis Adu-Amankwaah, Candice Februarie, Kudakwashe Nyambo, Gerald Maarman, Ndivhuwo Tshililo, Lawrence Mabasa, Vuyo Mavumengwana, Lucinda Baatjies

**Affiliations:** 1 South African Medical Research Council Centre for Tuberculosis Research, Division of Molecular Biology and Human Genetics, Faculty of Medicine and Health Sciences, Stellenbosch University, Cape Town, South Africa; 2 Biomedical Research and Innovation Platform (BRIP), Medical Research Council, Tygerberg, Cape Town, South Africa; 3 Division of Medical Physiology, Department of Biomedical Sciences, Faculty of Medicine & Health Science, CARMA: Centre for Cardio-Metabolic Research in Africa, Stellenbosch University, Cape Town, 8000 South Africa

**Keywords:** Triple-negative breast cancer, Cell death, *Eriocephalus racemosus*, Flow cytometry, Glycolysis, Mitochondrial activities, Liquid chromatography-mass spectrometry

## Abstract

**Introduction:**

Triple-negative breast cancer (TNBC) represents a significant global health crisis due to its resistance to conventional therapies and lack of specific molecular targets. This study explored the potential of *Eriocephalus racemosus* (*E. racemosus*) as an alternative treatment for TNBC. The cytotoxic properties and high-resolution respirometry mitochondrial activities of *E. racemosus* against the MDA-MB 231 TNBC cell line were evaluated.

**Methods:**

Hexane solvent and bioactive fraction extractions of *E. racemosus* were performed, while mass spectrometry-based metabolite profiling was used to identify the phytochemical constituents of the extracts. The extracts were further tested against MDA-MB 231 TNBC cells to determine their cytotoxicity. The mode of cell death was determined using flow cytometry. The activities of caspases 3, 8, and 9 were assessed using a multiplex activity assay kit. Glycolytic activity and High-resolution respirometry measurements of mitochondrial function in the MDA-MB 231 cell line were conducted using the Seahorse XFp and Oroboros O2K.

**Results:**

Metabolite profiling of *E. racemosus* plant crude extracts identified the presence of coumarins, flavonoids, sesquiterpenoids, triterpenoids, and unknown compounds. The extracts demonstrated promising cytotoxic activities, with a half maximal inhibitory concentration (IC_50_) of 12.84 µg/mL for the crude hexane extract and 15.49 µg/mL for the bioactive fraction. Further, the crude hexane and bioactive fraction extracts induced apoptosis in the MDA-MB-231 TNBC cells, like the reference drug cisplatin (17.44%, 17.26% and 20.25%, respectively) compared to untreated cells. Caspase 3 activities confirmed the induction of apoptosis in both cisplatin and the plant crude extracts, while caspase 8 and 9 activities confirmed the activation of both the intrinsic and extrinsic apoptosis pathways. Increased levels of glycolytic activity were observed in the hexane crude extract. High-resolution respiratory measurements showed elevated mitochondrial activities in all mitochondrial states except for complex-IV activity.

**Conclusion:**

These findings support further exploration of *E. racemosus* as a potential therapeutic agent for TNBC, offering a promising avenue for the development of targeted treatments with minimal adverse effects.

**Supplementary Information:**

The online version contains supplementary material available at 10.1186/s12906-024-04615-x.

## Introduction

In the complex world of medical research, one major challenge that has always posed a formidable threat to scientists and healthcare practitioners is triple-negative breast cancer (TNBC). This non-communicable malignancy strikes at the very core of human physiology, transcending geographical boundaries and impacting individuals across the spectrum, making it a global health crisis [1, 2]. Thus, TNBC poses a significant challenge to oncologists and scientists worldwide [3]. Existing therapeutic approaches have failed to provide adequate solutions to effectively treat this affliction, highlighting the need for innovative interventions. The ineffectiveness of traditional hormone-based and targeted therapies stems from the fact that specific molecular targets remain undiscovered in TNBC, leaving researchers and clinicians searching for more successful alternatives [3, 4]. Moreover, most of the current chemotherapy approaches lack specificity and selectivity, which often ends up impacting cancerous and healthy cells without discrimination [4].

Mitochondria, which are often referred to as the “powerhouses” of the cell due to their crucial role in generating cellular energy in the form of adenosine triphosphate (ATP), not only fulfil this vital function but also serve as a central hub for a plethora of cellular processes [5, 6]. The dysregulation of mitochondrial function has been extensively associated with the development of cancer, and the breast is certainly not exempt from this phenomenon. Traditionally, the Warburg effect has dominated discussions surrounding cancer metabolism, emphasising the reliance of cancer cells on glycolysis [7, 8]. However, recent discoveries have shed light on the fact that cancer cells are not strictly dependent on glycolysis and possess metabolic plasticity that enables them to adapt in the face of various stimuli, including pharmacological treatments [9]. The accumulation of emerging evidence from a diverse range of experimental models and tumour types has given rise to the hypothesis that a specific subset of quiescent or slow-cycling tumorigenic cells may share a common metabolic program that heavily relies on mitochondrial respiration rather than glycolysis [10, 11]. This metabolic program, which has adapted to the reduced anabolic demands of dormant cells, is of great interest due to its unique nature. In contrast, actively proliferating cancer cells require a delicate balance between mitochondrial respiration and glycolysis to sustain the constant supply of high-energy molecules and intermediary metabolites necessary for the biosynthetic pathways essential for their unremitting growth [5]. This hypothesis posits that a critical subset of cells responsible for tumour maintenance, metastasis and relapse following treatment may exhibit this mitochondrial-driven metabolic program across various cancer types. The implications of such a metabolic program extend far beyond cancer cell biology, as it opens new avenues for understanding the complex interplay between cellular energetics and disease progression [9, 12].

Considering these challenges, natural compounds have emerged as a promising avenue to tackle this pressing issue [13]. Among these natural products, fynbos and other South African plant species, particularly *Eriocephalus racemosus* (*E. racemosus*), have garnered attention due to their potential for therapeutic applications [14]. Recent investigation has revealed that extracts derived from *E. racemosus* may possess various properties, including potential anticancer activities [14]. Although research on the applications of *E. racemosus* for TNBC is still in its infancy, the plant’s multifaceted nature presents an exciting prospect for the development of treatments with minimal adverse effects.

There remains a significant gap in the existing body of literature concerning *E. racemosus*, and its assessment of cytotoxic characteristics [14]. Consequently, the present study aims to evaluate the in-vitro cytotoxic, glycolytic and high-resolution respirometry mitochondrial activity attributes of *E. racemosus* against the MDA-MB 231 TNBC.

## Materials and methods

### Plant collection and solvent extraction

The *E. racemosus* plant (voucher specimen number: 2951) was procured in the Overberg District, Western Cape, South Africa, from the Grootbos Private Nature Reserve (GPNR, 34.540°S 19.413° E) in February 2023. The authenticity of the plant was confirmed by Paula Strauss, an ecologist with the Grootbos Foundation. The collected plants were transported to the laboratory (Stellenbosch University, Tygerberg Campus) within twenty-four hours and left to air-dry for approximately 14 days at room temperature. The dried leaves were then ground into fine powders and stored at room temperature. Subsequently, 10 g of plant powder was weighed and immersed in 250 mL of organic solvent (hexane) (Servochem, South Africa) for metabolite extraction. The mixture was kept under constant shaking at 100 rpm overnight. To ensure complete extraction of metabolites, this process was repeated twice with hexane. The filtrate obtained after filtration of the plant powder-solvent mixtures was concentrated using a rotary evaporator (Buchi Rotavapor R-100, Switzerland) at 45 °C for hexane. The crude extract was stored at 4 °C for further downstream analysis, as described by Adu-Amankwaah et al., 2022.

### Determination of the cytotoxic activities of *E. racemosus* against MDA-MB 231 and vero cell lines

The TNBC cells (MDA-MB 231) and normal monkey kidney cells (Vero cells) used in this experiment were obtained from Prof Anna-Mart Engelbrecht at Stellenbosch University, South Africa and Prof Maryna van de Venter from Nelson Mandela University, South Africa, respectively. The cells were cultured, subcultured and maintained with Dulbecco’s modified Eagle’s medium (DMEM) supplemented with 10% fetal bovine serum (FBS) (complete DMEM). The cells were seeded in a 96-well plate at a density of 6 000 cells/200 µL per well and allowed to attach for 24 h before treatment. The plant crude extract was dissolved in dimethyl sulfoxide (DMSO) to form a 100 mg/mL stock. The cells were treated with plant crude extract at 7.8125, 15.625, 31.25, 62.5, 125 and 250 µg/mL and cisplatin (Sigma Aldrich, USA) at 3 µg/mL (as the reference drug). Complete DMEM was used to culture the cells and was replaced with 0.5 mg/mL 3-(4,5-Dimethylthiazol-2-yl)-2,5-diphenyltetrazolium bromide (MTT) after 48 h of treatment. The plates were incubated for an additional four hours, after which the MTT solution was removed, and the formazan product was dissolved in 100 µL DMSO. The absorbance was measured at 540 nm using a microtiter plate reader (FLUOstar Omega, Germany). All incubations were performed in a humidified incubator with 5% CO_2_ at 37 °C (ESCO, Vivid Air). The experimental method was performed as previously described with minor modifications, as outlined by Adu-Amankwaah et al., 2022 [14, 15–17].

### Mode of cell death (Annexin VFITC/PI apoptosis assay)

The Annexin V-FITC/Propidium Iodide (PI) apoptosis assay was conducted on MDA-MB 231 TNBC cells that were seeded in 24-well plates at a density of 2.5 × 10^5^ cells/well. The cells were then incubated overnight at 37 °C in a humidified incubator with 5% CO_2_ (ESCO, Vivid Air). Afterwards, the cells were treated with cisplatin (10 µM/3 µg/mL) and the plant crude extract at their respective IC_50_ values for 48 h. Following incubation, 1 mL of 1× Trypsin (Sigma, USA) (diluted in PBS) was added to detach the cells. The cells were allowed to recover in a complete medium for an hour. After recovery, the cells were transferred to polypropylene flow cytometry tubes and harvested through centrifugation at 1500 rpm for 5 minutes at 4 °C. The pellets were washed with ice-cold complete DMEM media. The Annexin V-FITC/PI apoptosis detection kit was utilised to stain the cells according to the manufacturer’s instructions (Invitrogen, Thermo Fisher Scientific). Briefly, the pellets were redissolved in ice-cold 1× binding buffer. Annexin V-FITC and PI were added to each tube, 1 µL and 5 µL, respectively. Control tubes with single stains were also added and incubated in the dark for 15 min. After incubation, 400 µL of 1 × annexin-binding buffer was added and gently mixed. The samples were read on a BC DxFlex flow cytometer (Beckman Coulter, USA) and a fluorescent microscope (Zeiss, Germany) [14, 15].

### Determination of caspase 3, 8 and 9 activities of the *E. racemosus*

Caspases 3, 8 and 9 activities were determined using a multiplex activity assay kit (Fluorometric, Abcam, USA). MDA-MB-231 TNBC cells were plated at a density of 2 × 10^4^ cells/90 µL in a 96-well plate and maintained in a 5% CO_2_ humidified incubator at 37 °C overnight. To measure the activities of Caspase 3, 8, and 9, cells were treated with plant crude extract (IC_50_ values) and cisplatin (3 µg/mL) for 48 h. The manufacturer’s instructions were followed to prepare the assay loading solutions for each caspase substrate. This involved adding 50 µL of the respective substrate to 10 mL of assay buffer and mixing the mixtures thoroughly by pipetting up and down. Subsequently, 100 µL of the respective Caspase assay (3, 8 and 9) loading solution was added directly to each well without removing the culture media or treatment solution. The plates were incubated for four hours at 37 °C and 5% CO_2_. A fluorescence microplate reader (FLUOstar Omega, Germany) was set up with either bottom read mode and configured to measure each caspase’s specific excitation and emission wavelengths. The excitation and emission wavelengths for Caspase 3, Caspase 8, and Caspase 9 were 535/620 nm (red), 490/525 nm (green), and 370/450 nm (blue), respectively [18].

### Assessment of glycolysis in MDA-MB 231 triple-negative breast cancer cell

To assess the impact of plant crude extracts on the glycolytic activity of MDA-MB 231 TNBC cells, measurements were conducted using the Agilent Seahorse Bioscience XFp Extracellular Flux Analyzer, following the manufacturer’s guidelines as delineated in the Agilent XF manual (Glycolysis Stress Test assay). Briefly, cells were seeded in XFp Cell Culture Microplates (Seahorse Bioscience) at a density of 2 × 10^4^ cells/well and allowed to incubate for 1 h in XF DMEM medium devoid of phenol red, supplemented with 210 mM glucose, 2 mM sodium pyruvate, and 2 mM glutamine. Basal rates were recorded over three measurement intervals. Subsequently, mitochondrial electron transport chain inhibitors, Rotenone and Antimycin A (Rot/AA), were introduced to suppress mitochondrial oxygen consumption, thereby inhibiting CO_2_-derived protons. Following this, a subsequent injection of 2-deoxy-D-glucose (2-DG), a glucose analogue, was administered. 2-DG functions by competitively binding to glucose hexokinase, the initiating enzyme in the glycolytic pathway, thereby inhibiting glycolysis. The resultant reduction in proton efflux rate (PER) served as qualitative evidence that the PER generated before injection predominantly originates from glycolysis. Subsequent data analysis involved normalising result values to cell numbers for accurate interpretation [19].

### Mitochondria activity of MDA-MB 231 cell line when treated with *E. racemosus* (high-resolution respirometry using the oroboros oxygraph-2 K)

Mitochondrial activity of MDA-MB 231 TNBC cells was measured using a modified method by Kriel et al., 2018. Mitochondrial respiration was measured through high respiration respirometry using the Oroboros O2K (Oroboros Instruments, Innsbruck, Austria). The amplified signal generated by the oxygen sensor was recorded on a computer, utilising the DatLab acquisition software (Oroboros Instruments, Innsbruck, Austria). The cells were washed with pre-warmed PBS after incubation at 37 °C for 48 h following treatment. The cells were detached using 0.25% trypsin and centrifuged at 1300 rpm for 5 min. The cell pellet was resuspended in mitochondrial respiration (MIR05) medium [20], adjusted to concentrations ranging from 1.5 × 10^6^ to 2.5 × 10^6^ cells/mL, and transferred to chambers A and B, respectively. The SUIT-001_O2_ce-pce_D003 protocol was chosen for the substrate-uncoupler Inhibitor-Titration experiment (SUIT) [21]. Prior to the experiment, pure oxygen of 500–600 µM was added to the chambers to achieve sufficient oxygen to avoid oxygen depletion and undesired limitations on mitochondrial oxygen consumption. Untreated cells (MDA-MB 231), cells treated with the reference drug (cisplatin, 3 µg/mL), and cells treated with *E. racemosus* (IC_50_) were added to the chambers.

The SUIT-001_O2_ce-pce_D003 protocol involved the addition of various substances in a specific sequence. The first step was the addition of digitonin (Dig), which caused cell permeabilisation at a concentration of 16.0 µg/mL. The second step involved the addition of pyruvate (P) (5 mM) and malate (M) (2 mM) to determine NADH-linked (complex I-linked) leak respiration (LR). The third step stimulated complex I-linked oxidative phosphorylation (C-I) by adding adenosine diphosphate (ADP) (1 mM). The fourth step involved adding cytochrome C (c) (Cyt-C) (10 µM) to assess the integrity of the outer mitochondrial membrane (OMM). The fifth step included stepwise titrations of the uncoupler carbonyl cyanide m-chlorophenylhydrazone CCCP (U) (0.5 µM) to determine noncoupled CI-linked respiration, corresponding to electron transfer system capacity (ETS capacity) with CI-linked substrates.

The sixth step consisted of glutamate (G) (10 mM) which forms part of the LR (together with P and M) to stimulate complex I-linked OXPHOS to a maximal level. The seventh step involved the addition of succinate (S) (10 mM) to support electron flow from the C-II-pathway into the Q-junction (representing the ETS capacity of the C-I&II-pathway). The eighth step entailed titration of octanoylcarnitine (Oct) (0.5 µM) to stimulate respiration via the simultaneous action of the F-pathway, N-pathway, and S-pathway. The ninth step included adding the C-I inhibitor rotenone (Rot) (1 µM) to inhibit electron flow from the C-I pathway and measure the C-II-linked ETS capacity. The tenth step involved the addition of complex III (C-III) inhibitor antimycin A (2.5 µM) (Ama) to inhibit mitochondrial respiration and detect residual oxygen consumption (Rox) due to oxidative side reactions.

The eleventh step entailed adding Ascorbate (As) (2 mM) and tetramethyl-phenylenediamine (TMPD) (Tm) (0.5 mM), where TMPD is a C-IV-specific electron donor, and ascorbate ensures the reduction of TMPD, enabling a linear rate of C-IV activity. This step was followed by the addition of sodium azide (Azd) (200 mM) to provide the true complex IV activity. This protocol was chosen because it provided information on mitochondrial respiratory control and assesses the electron transfer system’s functional integrity. The phosphorylation system control ratio (P/E ratio) was calculated as:

$$\:\frac{\text{C}omplex-I\:linked\:OXPHOS}{Electron\:transfer\:system\:capacity}$$

The PE ratio is an expression of the limitation of oxidative phosphorylation capacity by the phosphorylation system.

The Leak/OXPHOS (L/P) coupling-control ratio was also calculated as:

$$\:\frac{Complex-I\:linked\:leak\:respiration}{Complex-I\:linked\:OXPHOS}$$

The LP ratio provides valuable insights into the potential limitation of oxidative phosphorylation by leak respiration.

### Extraction of bioactive fractions

#### Gravity column chromatography

The hexane plant crude extract (800 mg) was separated by silica gel column chromatography. Afterwards, a stepwise gradient elution was performed with gradients of ethyl acetate (Servochem, South Africa) to hexane (various concentrations of 0-100% to 0–20%) and 100% methanol. The fractions were left to dry in the fume hood [22].

#### Bioassays of bioactive fractions

Bioassays were conducted according to the procedures outlined in Sect. 2.2, 2.3, 2.4, and 2.8 to assess the activity and tentatively identify the compounds in the bioactive fractions.

### Preliminary identification of phytochemical components

High-resolution ultra-performance liquid chromatography-mass spectrometry (UPLC-MS) analysis was conducted using a Waters Cyclic Quadrupole time-of-flight (qTOF) mass spectrometer coupled to a Waters Acquity UPLC system (Waters, Milford, MA, USA). The eluate from the chromatographic column was initially directed through a Photodiode Array (PDA) detector before entering the mass spectrometer, enabling simultaneous acquisition of UV and MS spectra. Electrospray ionisation was employed in negative ion mode with a cone voltage set at 15 V, a desolvation temperature of 275 °C and a desolvation gas flow rate set at 650 L/h, while other MS parameters were optimised to ensure optimal resolution and sensitivity. Data acquisition was performed by scanning from m/z 150 to 1500 in resolution mode, as well as in MSE mode, wherein two channels of MS data were acquired. The first channel operated at a low collision energy of 4 V, while the second channel utilised a collision energy ramp ranging from 40 to 100 V to facilitate fragmentation analysis. Leucine enkephalin served as the lock mass for accurate mass determination, and instrument calibration was performed using sodium formate. Chromatographic separation was achieved on a Waters HSS T3 column (2.1 × 100 mm, 1.7 μm) with an injection volume of 2 µL. The mobile phase comprised 0.1% formic acid (solvent A) and acetonitrile containing 0.1% formic acid (solvent B). The gradient elution program was initiated with 100% solvent A for 1 min, followed by a linear gradient to 28% solvent B over 15 min. Subsequently, solvent B was increased to 40% over 50 s, followed by a 1.5 min wash step with 100% solvent B and re-equilibration to initial conditions over 4 min. The flow rate was maintained at 0.3 mL/min and the column temperature was set at 55 °C. [14, 23].

Subsequently, the acquired raw data containing spectral information were converted into .abf format. The .abf files underwent processing through the employment of MS-Dial module (version 4.24) and MS-Finder (version 3.5) software tools for preliminary identification of compounds. Parameters for this identification included an error threshold of ppm < 7.0 and the presence of [M + H]^+^ adduct ions. Additionally, manual annotation of compounds was undertaken using the KNapSacK (http://www.knapsackfamily.com/KNApSAcK_Family/), natural compound library (http://prime.psc.riken.jp/compms/msdial/download/msp/MSMS-Pos-Vaniya-Fiehn_Natural_Products_Library_20200109.msp) and Metfrag (https://msbi.ipb-halle.de/MetFrag/) compound databases.

### Statistical analysis

All grouped data were statistically analysed using Microsoft Excel 2010 and Graph Pad Prism version 8. Student’s t-tests and multiple t-tests were used for the hypothesis testing where a *p*-value of less than 0.05 was considered statistical significantly different.

## Results

### Evaluation of the cytotoxicity activity of *E. racemosus* crude extract and bioactive fractions against MDA-MB 231 cells

**Fig. 1:**
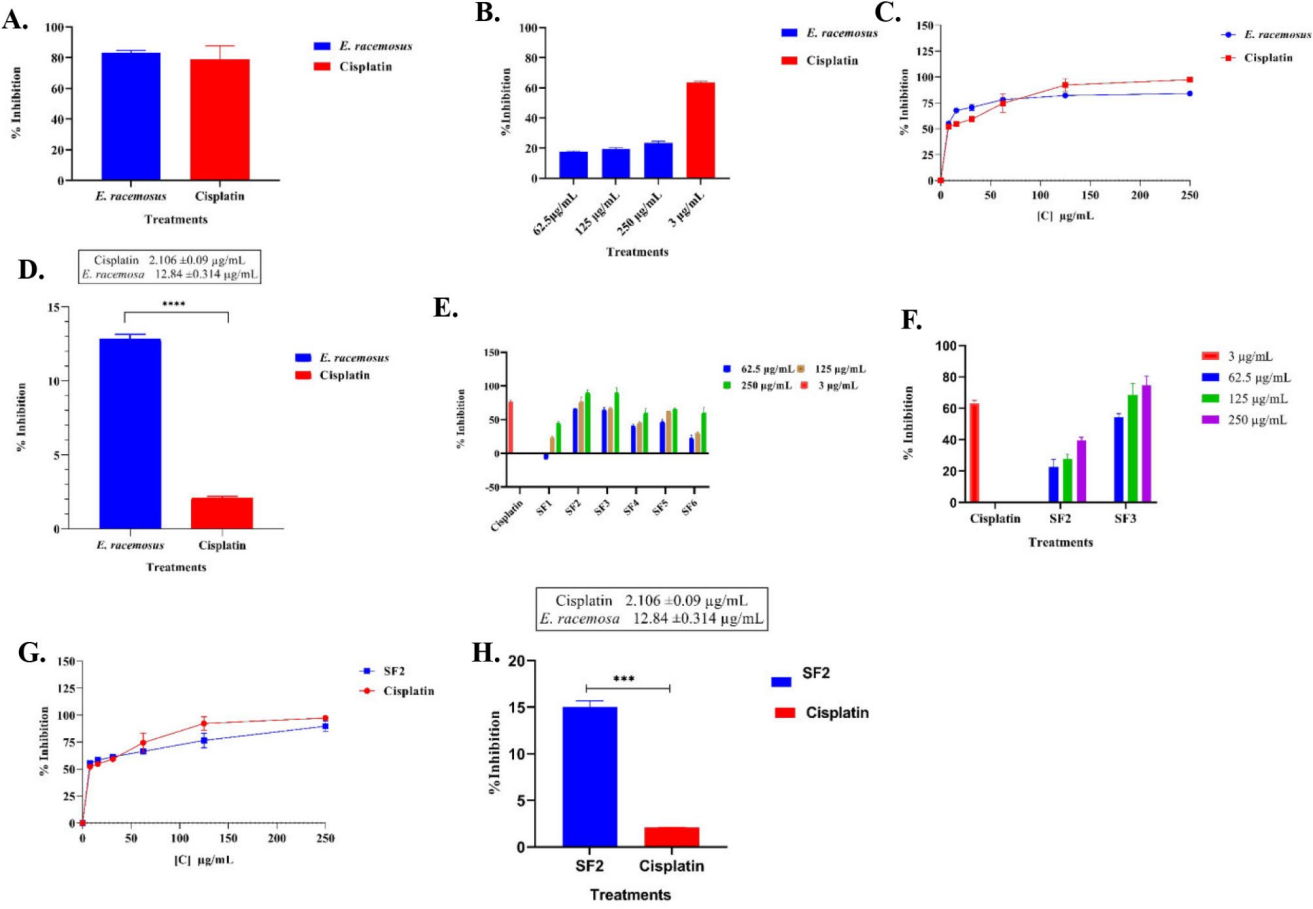
Anti-proliferative activity of *E. racemosus* leaf crude extract, bioactive fractions and cisplatin as reference drug against TNBC. (**A**) Initial cytotoxicity screen of *E. racemosus* leaf crude extract against MDA-MB 231 TNBC cells. (**B**) Cytotoxic activity of *E. racemosus* and cisplatin against Vero cells line; (**C**) Dose-response curves were constructed to assess the cytotoxicity of *E. racemosus* and cisplatin against MDA-MB 231 TNBC cells; (**D**) IC_50_ of the crude leave extracts of *E racemosus*; (**E**). Initial cytotoxicity screen of *E. racemosus* bioactive fraction extracts against MDA-MB 231 TNBC cells; (**F**) Cytotoxic activity of the selected bioactive fractions of *E. racemosus* (SF2) and cisplatin against Vero cells lines; (**G**) Dose-response curves of the 80% ethyl acetate and 20% hexane of *E. racemosus* fraction extract and cisplatin against MDA-MB 231 TNBC (**H**) IC_50_ of the crude leave extracts SF2. Results represent the mean ± SD of three technical and biological replicates

An initial pre-screen of the cytotoxic effect of *E. racemosus* leaf crude extract against the MDA-MB 231 TNBC cells was done. *E. racemosus* leaf crude extract was screened at 250 µg/mL, while cisplatin (reference drug) was screened at 3 µg/mL. The plant crude extract showed growth inhibition of 83.40% when screened against MDA-MB 231 cells compared to cisplatin, which showed 78.97% growth inhibition (Fig. 1A and Supplementary Fig. 1).

To ensure that the leaf crude extract did not cause any adverse effects on normal cells while potentially exhibiting anti-proliferative activity against cancer cells, the cytotoxic effects of the extract were evaluated against Vero cells, a normal African monkey kidney cell line control. Cisplatin (3 µg/mL) showed growth inhibition of 62.55% against the Vero cells highlighting its cytotoxic effect against the normal cell line control (Fig. 1B). *E. racemosus* leaf crude extract showed growth inhibition of 17.65%, 19.30% and 23.54% when screened against the Vero cells at 62.5 µg/mL, 125 µg/mL, and 250 µg/mL, respectively (Fig. 1B). Importantly, these values remained below the critical threshold of 50%, indicating that *E. racemosus* leaf crude extracts did not exert significant cytotoxic effects against the Vero cells at the tested concentrations.

The concentration at which *E. racemosus* leaf crude extract inhibits 50% of MDA-MB-231 cell growth (IC_50_) was determined. The plant crude extract was screened at concentration range of 7.8125, 15.625, 31.25, 62.5, 125 and 250 µg/mL, while cisplatin was assessed across the concentration range of 0.9375, 1.875, 3.75, 7.5, 15, and 30 µg/mL (Fig. 1C and Supplementary Table 1). The IC_50_ value of *E. racemosus* leaf crude extract was 12.84 ± 0.314 µg/mL. Cisplatin, a well-established reference drug, exhibited a significantly lower IC_50_ concentration of 2.106 ± 0.09 µg/mL. The results indicate that cisplatin was more potent than the crude extract in inhibiting the proliferation of MDA-MB-231 TNBC cells. However, the extract still exhibited a significant level (*p*-value < 0.0001) of anti-proliferative activity, which suggests that it has potential as a treatment option (Fig. 1C-D and Supplementary Table 1). This approach aligns with the guidelines set by the American National Cancer Institute (NCI) for the 50% inhibition limit characterisation of crude extracts. The NCI guidelines recommend using a 30 µg/mL threshold to identify extracts that warrant further investigation for potential anticancer properties [24, 25].

The six fractions of *E. racemosus* leaf crude extracts (Supplementary Table 2) were also screened against the TNBC cells at concentrations ranging from 62.5 to 250 µg/mL, cisplatin was screened at 3 µg/mL. Fractions SF2 (80% ethyl acetate and 20% hexane) and SF3 (60% ethyl acetate and 40% hexane) showed the highest growth inhibition of 89.76% and 89.89%, respectively, when screened at 250 µg/mL. SF1 (methanol) showed the lowest inhibition of 44.59% (Fig. 1E).

The cytotoxic profile of SF2 and SF3 was evaluated against the Vero cells. SF2 showed growth inhibition of 24.68%, 31.03%, and 39.56% at 62.5 µg/mL, 125 µg/mL, and 250 µg/mL, respectively. SF3 exhibited notable toxicity, with growth inhibition rates of 54.27%, 68.45%, and 77.89% at the same concentrations. SF2 values remained below the critical threshold of 50%, indicating minimal cytotoxicity against Vero cells within the tested concentration range (Fig. 1F).

The 80% ethyl acetate and 20% hexane fraction (SF2) extract was selected for further screening and its IC_50_ was determined using concentrations ranging from 7.8125 to 250 µg/mL. Cisplatin was evaluated at a concentration range from 0.9375 to 30 µg/mL (Fig. 1G and Supplementary Table 3). The IC_50_ of the *E. racemosus* bioactive fraction (SF2) was determined to be 15.49 ± 0.950 µg/mL. Once again, cisplatin showed a lower IC_50_ value of 2.106 ± 0.09 µg/mL, as was observed with the plant crude extract (Fig. 1G-H and Supplementary Table 3).

### Determination of mode of cell death using annexin-V and propidium iodide (PI) staining

**Fig. 2:**
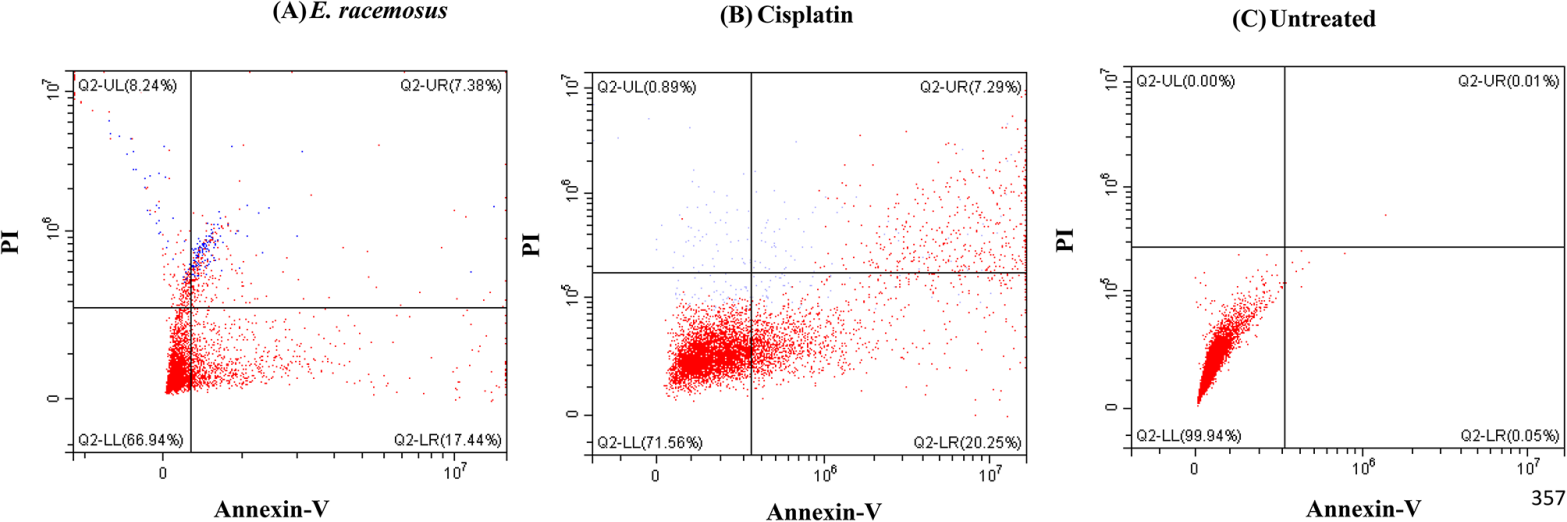
Flow cytometry analysis of the mode of cell death (apoptosis vs. necrosis) of MDA-MB 231 TNBC cells. (**A**) Untreated control cells, (**B**) Cisplatin reference control at 3 µg/mL and (**C**) *E. racemosus* plant crude extracts at 12.84 µg/mL. A minimum of 30 000 events were recorded for each sample. The experiment was done in triplicate and a representative plot of each treatment is shown

The mode of cell death in response to treatments with *E. racemosus* leaf crude extract and cisplatin was evaluated using the Annexin-V/PI kit from Invitrogen (ThermoFisher Scientific) via flow cytometry. Annexin-V renowned for its remarkable specificity towards phosphatidylserine (PS) residues, has emerged as an exceptional marker for apoptosis, a physiological process characterised by programmed cell death. This specific protein possesses the capacity to avidly bind to PS molecules, particularly when the structural integrity of the plasma membrane has been compromised, ultimately leading to the externalisation of PS from the inner to the outer cell membrane [26]. Conversely, PI, a highly efficient and selective DNA-binding fluorescent dye, exhibits the remarkable ability to penetrate solely into cells that have succumbed to death, specifically those with compromised plasma membranes [26]. In the flow cytometer evaluation, cells were categorised in the forward-side scatter plot as follows: those stained with both Annexin-V and PI were identified as late apoptotic cells in the upper-right (UR) region. Cells stained with Annexin-V alone were classified as early apoptotic cells in the lower-right (LR) region. Cells stained with PI alone were designated as necrotic cells in the upper-left (UL) region, and cells not stained with either were considered live cells in the lower-left (LL) region (Fig. 2).

**Fig. 3:**
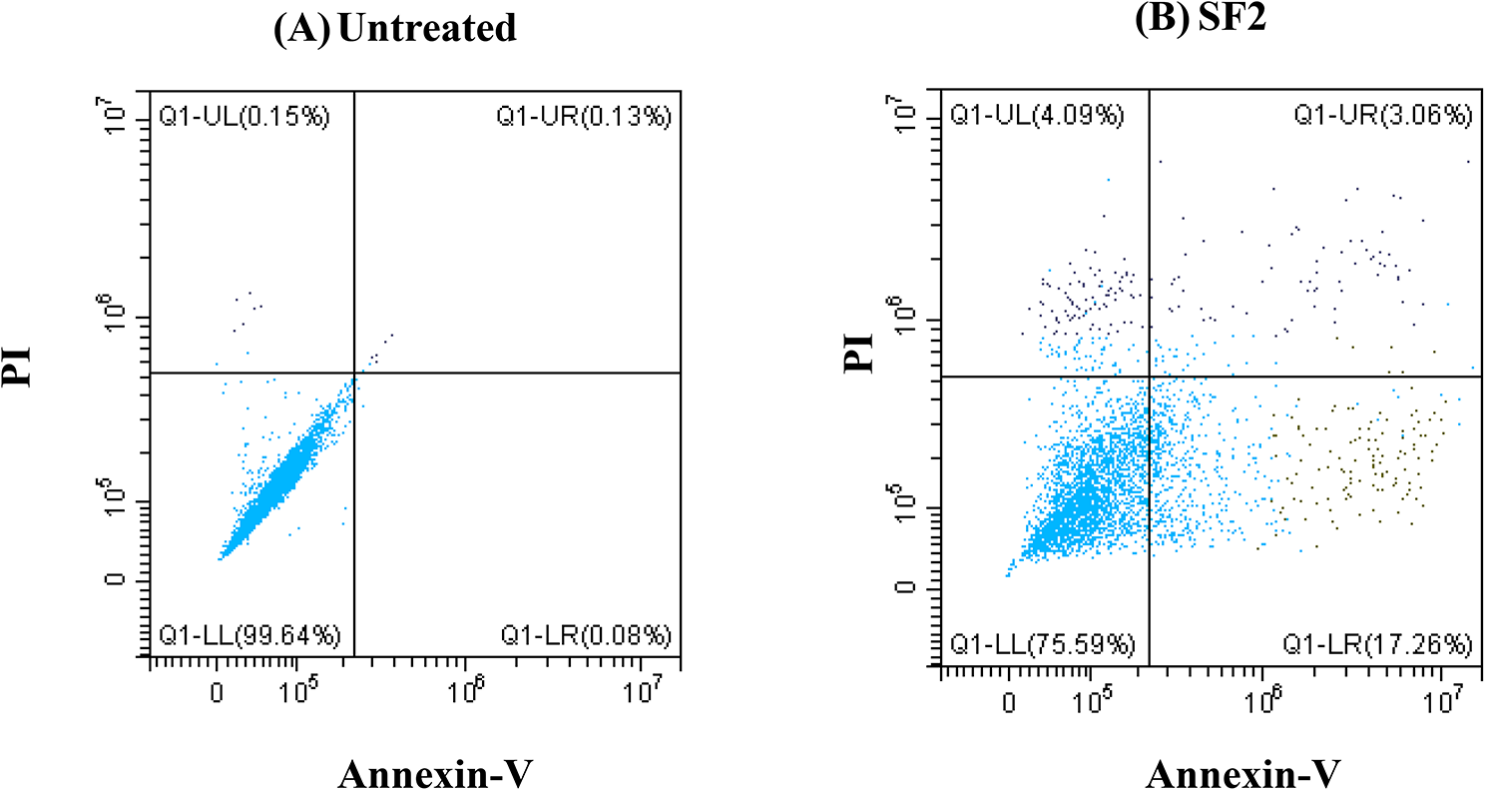
Flow cytometry analysis of the mode of cell death (apoptosis vs. necrosis) of MDA-MB 231TNBC cells. (**A**) Untreated control cells, (**B**) SF2, *E. racemosus* bioactive fraction at 15.49 µg/mL. A minimum of 30 000 events were recorded for each sample. The experiment was done in triplicate and a representative plot of each treatment is shown

Cisplatin and *E. racemosus* leaf crude extracts induced 20.25 ± 1.43% and 17.44 ± 0.97% apoptosis and 0.89 ± 1.02% and 8.24 ± 1.12% necrosis, respectively, when compared to the untreated cells, that showed 0.05 ± 1.10% apoptosis and 0.00 ± 0.19% necrosis (Figs. 2 and 3). The study’s findings specifically demonstrated that the condition screened *E. racemosus* leaf crude extracts induced a similar increase in apoptosis as cisplatin. However, a higher percentage of necrosis was observed compared to cisplatin and the untreated control.

Similarly, the bioactive fraction (SF2) was assessed to see if it would induce a higher percentage of apoptosis than the leave crude extract. The study’s findings showed that SF2 induced 17.26 ±0.80% early apoptosis and 4.09 ±0.92% necrosis, similar to the crude extract under the condition screened (Fig. 3).

**Fig. 4:**
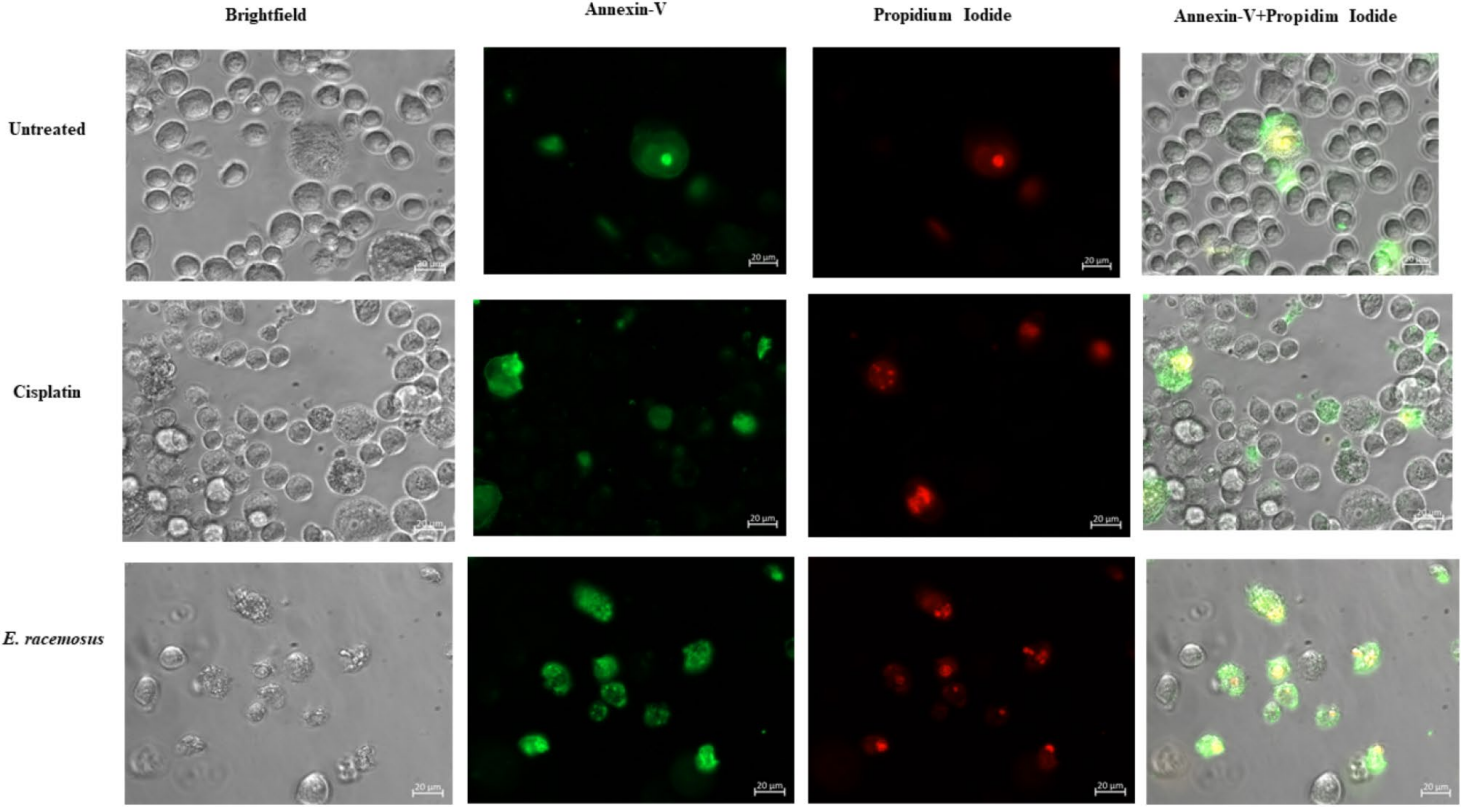
Fluorescent microscopy images of MDA-MB 231 TNBC stained with Annexin V and PI. MDA-MB 231TNBC cells were exposed *to* 3 µg/mL cisplatin and 12.84 ug/mL *E. racemosus* at a concentration of 12.84 ug/mL for 48 h. The cells were examined for apoptosis or necrosis using a fluorescent imaging microscope (Zeiss, Germany)

A parallel analysis was conducted for fluorescence microscope observations, where cells were stained with Annexin-V (green) or PI (red). Late apoptotic cells were evaluated as merging images captured using green and red filters (yellow signal) (Fig. 4). This was done to confirm the presence of apoptosis and necrosis observed in the flow cytometry assay.

### Validation of apoptosis by measuring caspase 3, 8 and 9 activities

Caspases are cysteine proteases and key enzymes in the apoptotic pathway. They play a crucial role in the initiation and execution of apoptosis. They can be broadly classified as signalling or effector caspases. Activation of Caspase 3 confirms the initiation of the execution phase of apoptosis, validating the early apoptosis detected by Annexin-V binding [27]. Caspases 8 and 9 are involved in initiating the apoptotic cascade, where caspase 8 is associated with the extrinsic pathway, triggered by external signals, while caspase 9 is involved in the intrinsic pathway, activated by internal cellular stress [28].

**Fig. 5:**
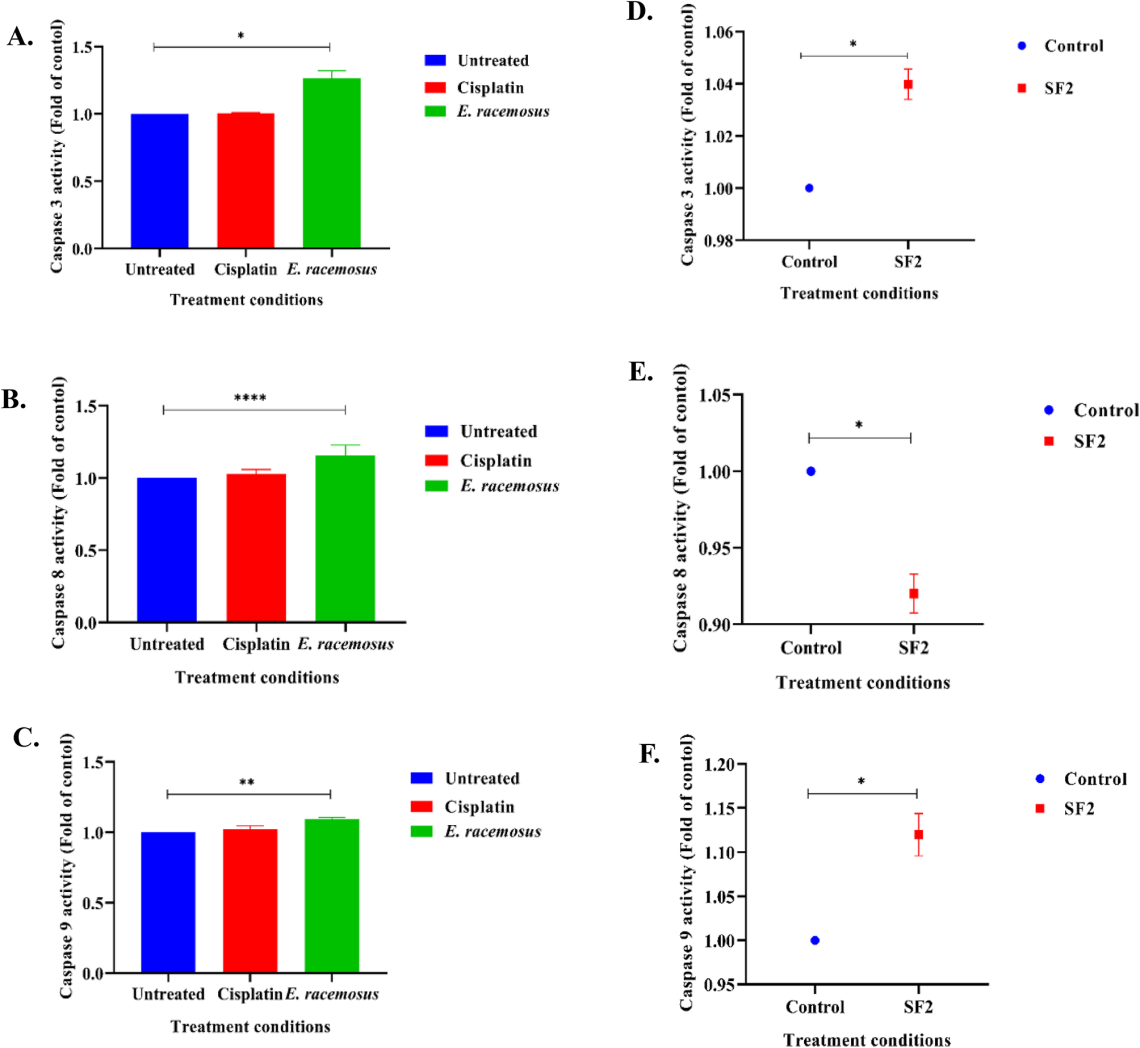
The effect of caspase activities of TNBC when untreated and treated with cisplatin and *E. racemosus* leaf crude extract, bioactive fraction and cisplatin. (**A**) Caspases 3 activity of the hexane crude leaves extract of *E. racemosus* and cisplatin; (**B**) Caspases 8 activity of the hexane crude leaves extract of *E. racemosus* and cisplatin; (**C**) Caspases 9 activity of the hexane crude leaves extract of *E. racemosus* and cisplatin; (**D**) Caspases 3 activity of the bioactive fraction (SF2) of *E. racemosus* leaf crude extracts; (**E**) Caspases 8 activity of the bioactive fraction (SF2); (**F**) Caspases 9 activities of the bioactive fraction (SF2). Activation of Caspase-3, 8 and 9 were expressed as folds of the control and was analysed using the GraphPad Prism 8 software. Results represent the mean ± SD of three technical and biological replicates. *, **, **** Indicate that the *p*-value is < 0.05, < 0.01 and < 0.0001 which is significantly different from the untreated control. The *p*-values were calculated using the two-tailed student t-test

This current study showed that caspase-3 activity was increased by 0.26-fold when treated with *E. racemosus* leaves crude extract compared to the untreated control (1.00-fold). Cisplatin, however, showed a lower increase in caspase-3 activity (1.01-fold) compared to the control group (Fig. 5A). The observed 1.26-fold increase in caspase-3 activity in TNBC cells validates the induction of early apoptosis in the cell line following treatment with *E. racemosus* leaves crude extract. Also, the present study provides elucidation regarding the impact of plant-derived bioactive fraction (SF2) on caspase-3 activity levels, demonstrating an increased fold change of 0.04 relative to the control (1.00-fold) (Fig. 5D).

The results suggest *E. racemosus* leaves crude extract significantly influences the activity of caspase 8, a pivotal player in the extrinsic apoptotic pathways. *E. racemosus* leaves crude extract induced a 0.20-fold increase, while cisplatin showed a slight increase of 0.03-fold in caspase 8 activity compared to the control (1.00-fold) (Fig. 4B). Interestingly, *E. racemosus* leaf crude extracts also showed higher caspase 9 activity 0.09-fold when compared to cisplatin and the untreated control, which showed 0.02 and 1.00-fold, respectively (Fig. 4C). These findings suggest that *E. racemosus* might influence the activation of caspases 8 and 9, potentially indicating its ability to induce apoptosis in the TNBC cells. The plant crude extract’s simultaneous activation of intrinsic and extrinsic apoptotic pathways suggests that it may be a versatile and useful candidate with implications for various medical diseases, especially those involving abnormal cell survival or proliferation.

The bioactive fraction SF2 showed a reduction in caspase 8 activity of 0.92-fold compared to the control, which showed 1.00-fold (Fig. 5E). SF2 showed an increase of caspase 9 activity of 0.20-fold compared to the control, which showed a baseline activity level equivalent to 1.00-fold (Fig. 5F). These findings suggest the bioactive fraction of *E. racemosus* might influence or induce apoptosis via the extrinsic apoptotic pathway.

### Evaluating glycolytic activity

Glycolysis, the metabolic pathway responsible for converting glucose into energy, takes on a unique significance. Cancer cells often exhibit increased rates of glycolysis, known as the Warburg effect, to meet their heightened energy demands and support rapid proliferation. This metabolic reprogramming not only fuels tumour growth but also contributes to the altered tumour microenvironment, making glycolysis a promising target for cancer therapy [10, 11]. Using the Agilent Seahorse XFp Analyzer in tandem with the Seahorse XFp Glycolytic Rate Assay enables precise quantification of glycolytic rates under basal conditions and compensatory glycolysis after mitochondrial inhibition. This assay effectively considers the contribution of CO_2_ to extracellular acidification resulting from mitochondrial/tricarboxylic acid (TCA) cycle activity, thereby yielding data directly comparable to lactate accumulation measurements. The proton efflux/basal glycolysis from viable cells encompasses both glycolytic and mitochondrial-derived acidification. The perturbation of mitochondrial function through the use of Rotenone and Antimycin A (Rot/AA) facilitates the quantification of mitochondrial-associated acidification. By subtracting mitochondrial acidification from the Total Proton Efflux Rate (PER), the Glycolytic Proton Efflux Rate is determined [19].

**Fig. 6:**
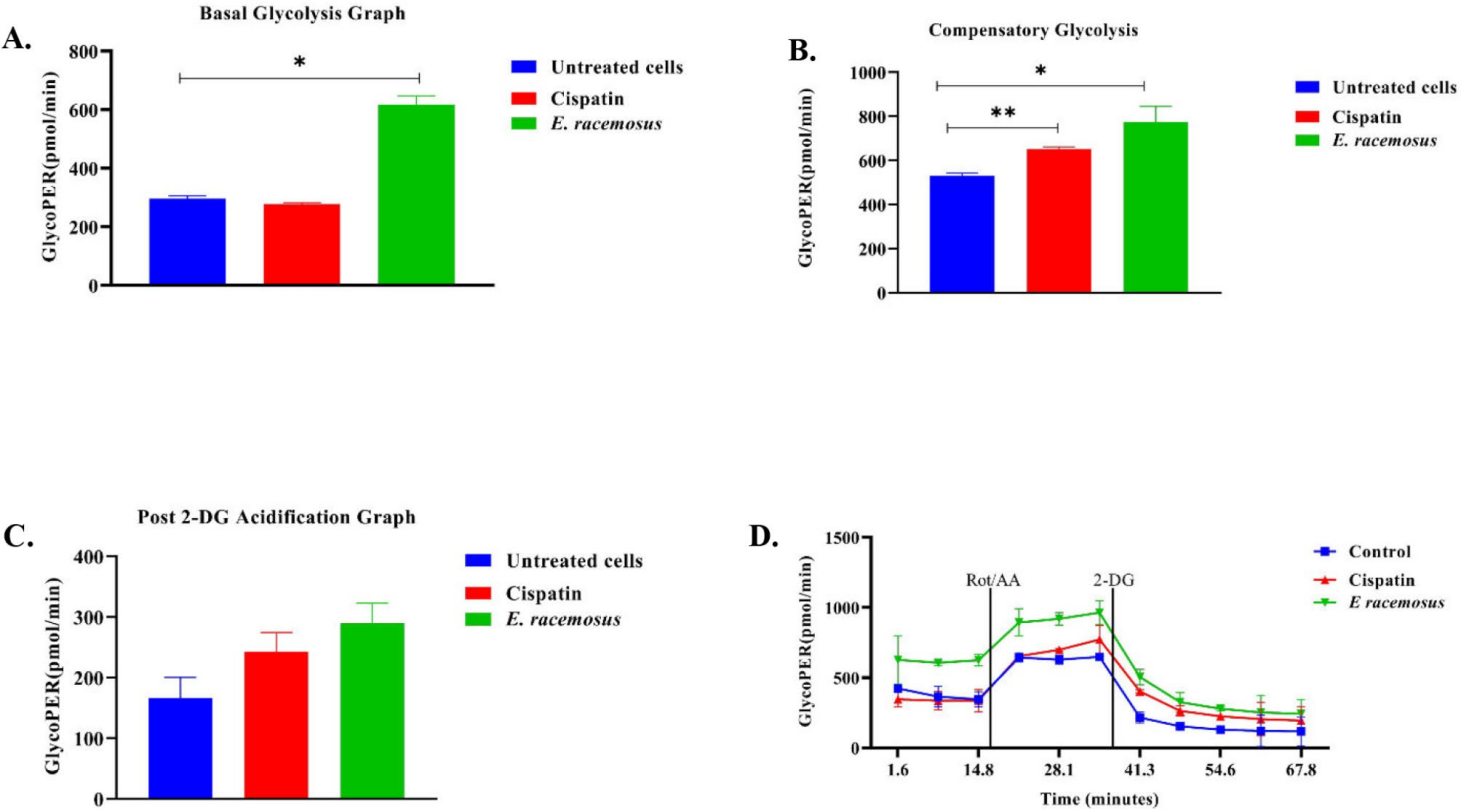
The metabolic effects of *E. racemosus* leaf crude extracts and cisplatin on MDA-MB TNBC cells. Four distinct aspects of glycolysis were evaluated: (**A**) Basal Glycolysis, (**B**) Compensatory Glycolysis, (**C**) Post 2-DG Acidification and (**D**) GlycoPER kinetic graph. The quantification of glycolytic impacts was performed relative to untreated cells using Agilent Seahorse Analytics and GraphPad Prism 8 software. Data were analysed from two independent experiments, each consisting of three technical replicates. Statistical significance (*p < 0.05 and **p < 0.001) compared to the untreated control was determined utilising the two-tailed Student’s T-test

The findings in this study showed a notable increase in basal glycolysis, compensatory glycolysis, and 2-DG acidification in TNBC cells after treatment with *E. racemosus* leaf crude extracts (616.82, 774.64, and 289.42 pmol/min, respectively) in comparison to untreated cells (295.84, 529.80, and 166.18 pmol/min, respectively). Conversely, cisplatin-treated cells exhibit increased levels of compensatory glycolysis and post-2-DG acidification (650.31 and 242.78 pmol/min, respectively) and a decreased level of basal glycolysis (277.78 pmol/min) in MDA-MB 231 TNBC cells relative to untreated cells (Fig. 6A-D). The increased compensatory glycolysis following treatment with *E. racemosus* leaves crude extract highlights a significant metabolic adaptation in TNBC cells. This compensatory glycolytic response, characterised by elevated glucose consumption and lactate production beyond baseline levels, highlights the metabolic reprogramming induced by the plant extracts. Interestingly, this phenomenon contrasts with the effects of cisplatin treatment, where compensatory glycolysis is similarly elevated but basal glycolysis is reduced. This divergence suggests distinct metabolic alterations elicited by different therapeutic agents, emphasising the potential of *E. racemosus* as a modulator of glycolytic metabolism in cancer cells (Fig. 6).

### Mitochondrial activity of MDA-MB 231 TNBC cells when treated with *E. racemosus* (high-resolution respirometry using the oroboros oxygraph-2 K)

In response to the high-resolution respirometry conducted to assess the mitochondrial activity of MDA-MB 231 TNBC cells, notable changes were observed, which have significant implications for cellular energy metabolism and overall oxygen consumption. An increase in routine respiration (RO) in cells treated with cisplatin (29.15 pmol·s^− 1^·mL^− 1^) compared to untreated cells (10.65 pmol·s^− 1^·mL^− 1^) was observed, and cells treated with *E. racemosus* leaf crude extracts showed an elevated level of routine respiration (20.46 pmol·s^− 1^·mL^− 1^). Both treatment groups demonstrated significantly higher leak respiration (LR) compared to untreated cells (control), with cisplatin-treated cells (64.06 pmol·s^− 1^·mL^− 1^) and *E. racemosus* leaf crude extract treated cells (67.77 pmol·s^− 1^·mL^− 1^) showing a considerable elevation in leak respiration, whereas untreated cells recorded a relatively lower value (17.75 pmol·s^− 1^·mL^− 1^).

Evaluation of the functional complexes within the mitochondria, Complex-I linked OXPHOS (C-I) showed a significant increase in both treatment groups, surpassing the levels observed in untreated cells. In addition, Cytochrome-C (Cyt-C) expression was increased in both cisplatin and *E. racemosus* leaf crude extract treated cells (34.29 pmol·s^− 1^·mL^− 1^ and 32.30 pmol·s^− 1^·mL^− 1^ respectively). The Electron Transfer System (ETS) and the respiration of Succinate dehydrogenase (SDH) also exhibited a noteworthy increase in both treatment groups, indicating an improved efficiency of the mitochondria. Conversely, β-oxidative linked OXPHOS (β-Ox) displayed only marginal changes. Interestingly, Complex-II linked respiration (C-II) demonstrated increased levels in cisplatin-treated cells (37.61 pmol·s^− 1^·mL^− 1^), further emphasising the alterations in mitochondrial activity.

**Fig. 7:**
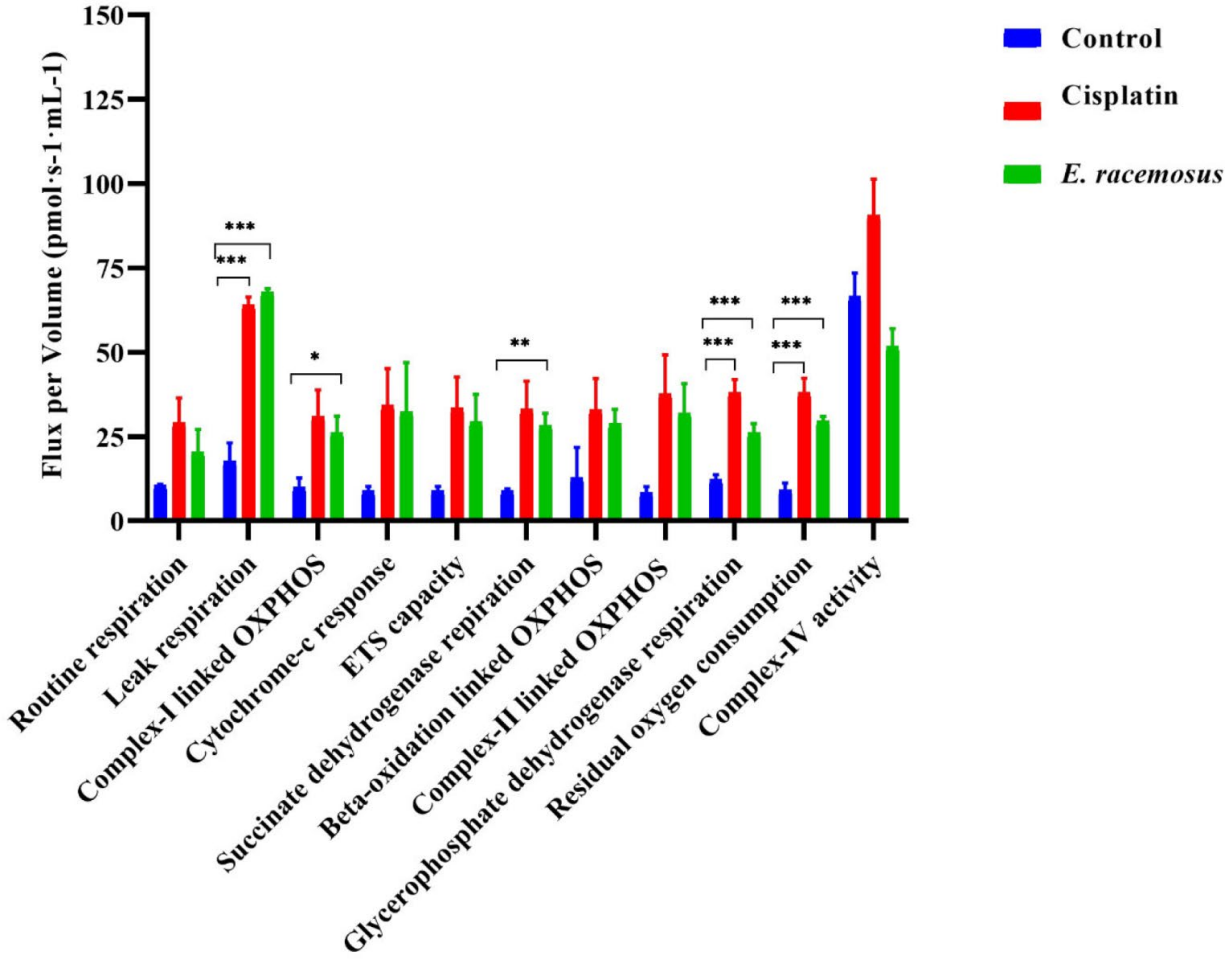
Mitochondrial bioenergetics of MDA-MB 231 TNBC cells when treated with cisplatin (3 µg/mL) and *E. racemosus* plant crude extract (13 µg/mL) compared to the untreated cells (control), as assessed through High-Resolution Respirometry (using Oroboros Oxygraph-2 K). The cellular respiration, represented as Flux per Volume, was examined at various stages (from routine respiration to complex-IV activity). The mean ± SD of triplicate experiments and the statistical significance were determined through multiple t-tests utilising the Holm-Sidak method (α = 0.05). Adjusted p-values were calculated and used in this experimental analysis, with significance levels denoted as follows: *p < 0.05, **p < 0.01, and ***p < 0.001

The results of the glycerophosphate dehydrogenase flux showed an increase in both treatment groups, with cisplatin-treated cells (37.98 pmol·s^− 1^·mL^− 1^) showing the most pronounced effect (*E racemosus*, 26.18 pmol·s^− 1^·mL^− 1^). Complex-IV (C-IV) showed increased activity in the cisplatin-treated cells (90.55 pmol·s^− 1^·mL^− 1^), while *E. racemosus* leaf crude extract (51.71 pmol·s^− 1^·mL^− 1^) showed a moderate effect when compared to the untreated cells (66.52 pmol·s^− 1^·mL^− 1^). These findings suggest a complex interplay between cisplatin, *E. racemosus* leaf crude extract, and mitochondrial activity within MDA-MB 231 TNBC cells (Fig. 7). The P/E ratio for the untreated group was 1.11 ± 1.28, indicative of the baseline mitochondrial phosphorylation capacity. In the presence of cisplatin and *E. racemosus* leaves crude extract, the P/E ratio showed a slight decrease of 0.93 ± 0.85 and 0.89 ± 0.60, respectively, implying a possible effect on the efficiency of mitochondrial oxidative phosphorylation. The leak/OXPHOS (L/P) coupling-control ratio also showed variations among the treatment groups. The untreated group showed an L/P ratio of 0.91 ± 1.35, signifying a basal level of mitochondrial leak respiration relative to oxidative phosphorylation. Cisplatin-treated cells showed a slight increase in the L/P ratio of 0.99 ± 1.11, suggesting a potential adaptation in response to the treatment. *E. racemosus* leaf crude extract treatment resulted in a significantly elevated L/P ratio of 1.48 ± 1.33, implying a distinct metabolic response with an increased leak respiration component (Table 1).

**Table 1:** Calculated control ratio and the coupling-control ratio

	Untreated (pmol·s^− 1^·mL^− 1^)	Cisplatin (pmol·s^− 1^·mL^− 1^)	*E. racemosus* (pmol·s^− 1^·mL^− 1^)
P/E ratio	1.11 ± 1.28	0.93 ± 0.85	0.89 ± 0.60
L/E ratio	0.91 ± 1.35	0.99 ± 1.11	1.48 ± 1.33

### Tentative identification of phytochemical components of the 80% hexane + 20% ethyl acetate bioactive fraction (SF2) obtained from *E. Racemosus*

**Fig. 8:**
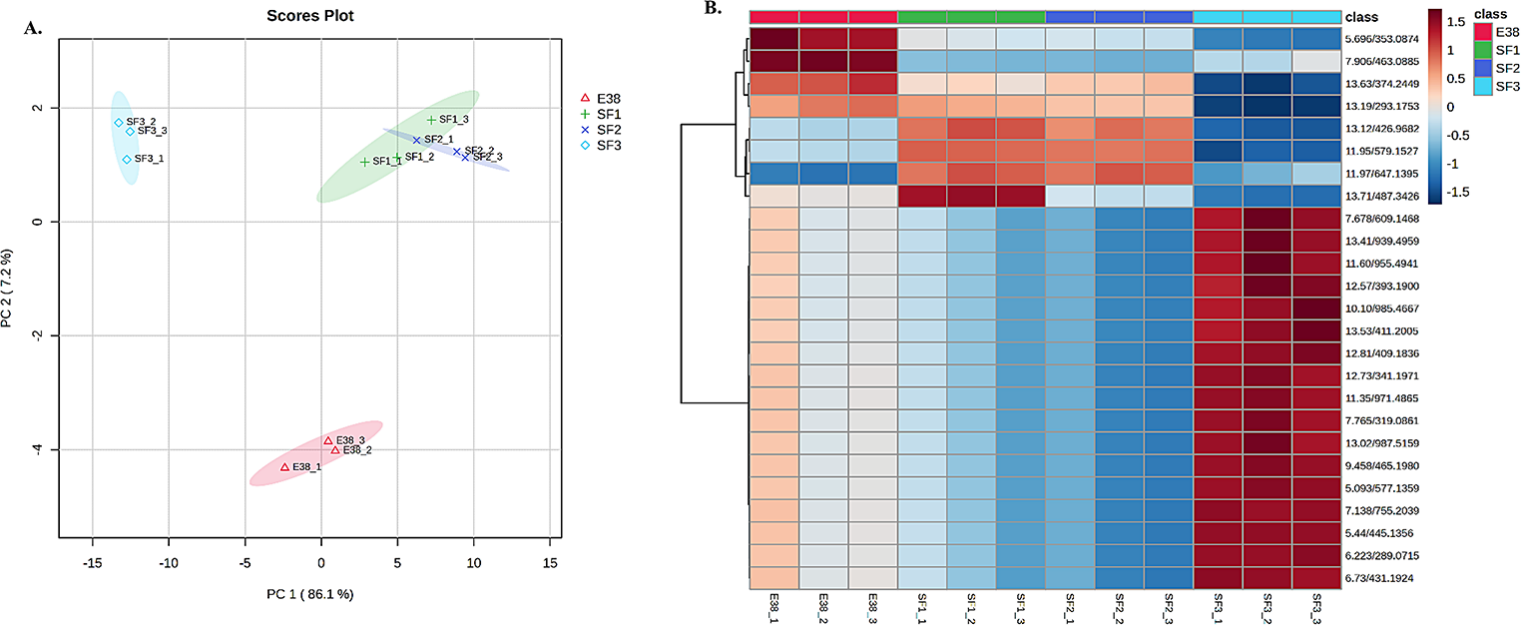
Principal Components Analysis (PCA) of the phytochemical component from *E. racemosus* leaf crude extract (E38), 100% Methanolic extract from *E. racemosus* (SF1), 80% Hexane + 20% ethyl acetate bioactive fraction from *E. racemosus* (SF2) and 60% Hexane + 40% ethyl acetate bioactive fraction from *E. racemosus* (SF3). (**A**) PCA scores plot comparing phytochemicals from crude extract (E38) and bioactive fractions (SF1, SF2 and SF3). (**B**) Heat map of the top 15 classes of phytochemicals from the crude extract (E38) and bioactive fractions (SF1, SF2 and SF3)

The chemical composition of the hexane crude extracts obtained from *E. racemosus* leave crude was examined using LC-QTOF-MS, facilitating the initial identification of various phytoconstituents. These compounds, which were tentatively identified, demonstrated a wide range of mass-to-charge ratio (m/z) values, indicating their diverse structural characteristics (Fig. 8; Table 2).

A comprehensive analysis of the compounds provisionally identified provides valuable insights into the wide range of phytochemicals present in *E. racemosus*. Among the identified constituents, the most notable classes include sesquiterpenoids, flavanoids and triterpenoids. It is worth noting that the extracts also contain several unidentified compounds, emphasising the potential for novel bioactive molecules within *E. racemosus*.

Some of the compounds tentatively identified and of particular interest include luteolin 7-glucuronide, a well-known plant-derived compound with potential bioactivity [29]. Furthermore, the extract also contained moronic acid, a plant triterpenoid known for its reported health benefits [30]. Hecogenin, a steroidal saponin recognised for its anti-inflammatory, antimicrobial and anticancer properties [30], further enhances the phytochemical profile (Fig. 8A-B; Table 2).

**Table 2:** Phytochemical composition of *E. Racemosus*

Compounds	Molecular formula	Ontology	RT (mins)	Precursor m/z	Peak Intensity	Mass Error (ppm)	Reference
**Hexane crude extract obtained from** *** E. racemosus***							
Unknown	-	-	13.20	293.1757	6668.2773	-	
Pechueloic Acid	C_15_H_20_O_3_	Sesquiterpenoid	14.89	249.1485	1509.7344	0.0843	Miski et al., [31]
Luteolin 7-glucuronide	C_21_H_18_O_12_	Flavonoids	7.73	463.0898	1765.3584	5.8261	Grass et al., [29]
2-(1-hydroxyethyl)-4-(2-hydroxypropyl)-2 H-furan-5-one	C_9_H_14_O_4_	Dihydrofurans	8.90	187.0972	1968	3.8216	Guo et al., [32]
Unknown	-	-	12.15	289.1053	3949.3516	-	
(2R,3R,4 S,5 S,6R)-2-[(2E)-4-ethenyl-2,5-dimethylhexa-2,5-dienoxy]-6-(hydroxymethyl)oxane-3,4,5-triol	C_16_H_26_O_6_	Fatty acyl	12.55	315.1810	1898.8145	2.4906	Robeson and Harborne, [33]
Unknown	-	-	14.71	357.2629	83006.625	-	
Dihydroalbocycline	C_18_H_30_O_4_	Macrolides and analogues	13.48	311.2224	1509.5964	0.0000	Gholami et al., [34]; Puri and Hall, [35]
Unknown	-	-	14.61	787.6255	69981.1875	-	
Cinerin II	C_21_H_28_O_5_	Fatty Acyls	14.41	361.1993	2714.3271	-4.5681	Harborne and Baxter, [36]
Moronic acid	C_30_H_46_O_3_	Triterpenoids	14.60	455.351	46262.3359	-2.1346	Matsuda et al., [37]
Colnelenic acid	C_18_H_28_O_3_	Fatty acyls	14.41	293.2118	12665.6289	2.315738	Galliard et al., [38]
Hecogenin	C_27_H_42_O_4_	Steroidal saponin	14.66	431.3145	4367.0068	-2.5179	Singh and Sharma, [30]
**80% Hexane + 20% ethyl acetate bioactive fraction (SF2) obtained from** *** E. racemosus***							
Luteolin 4’-O-glucoside	C_21_H_20_O_11_	Flavonoids	8.03	449.1100	11142.7979	4.8140	Grass et al., [29]
2-(1-hydroxyethyl)-4-(2-hydroxypropyl)-2 H-furan-5-one	C_9_H_14_O_4_	Furanones	8.91	187.0977	1724.0625	6.4940	Guo et al., [32]
Rhoifolin	C_27_H_30_O_14_	Flavonoids	11.96	579.1506	2671.8538	-6.2850	Abu-Reidah et al., [39]
(2R,3R,4 S,5 S,6R)-2-[(2E)-4-ethenyl-2,5-dimethylhexa-2,5-dienoxy]-6-(hydroxymethyl)oxane-3,4,5-triol	C_16_H_26_O_6_	Fatty Acyls	12.56	315.1811	1883.6326	2.8079	Clark et al., [40]
Moronic acid	C_30_H_46_O_3_	Triterpenoids	14.34	455.354	11628.9121	4.4537	Matsuda et al., [37]
Gypsogenic acid	C_30_H_46_O_5_	Triterpenoids	14.41	487.3433	63577.6328	3.0759	Gholami et al., [34]
Colladonin	C_24_H_30_O_4_	Coumarins and derivatives	14.48	383.2233	3875.4692	4.2117	Ahmed et al., [41]
Gypsogenin	C_30_H_46_O_4_	Triterpenoids	14.51	471.3451	22053.8398	-3.7891	Gholami et al., [34]
8-[3-oxo-2-[(E)-pent-2-enyl]cyclopenten-1-yl]octanoic acid	C_18_H_28_O_3_	Fatty Acyls	14.63	293.2100	3752.7969	-3.8232	Ye et al., [42]
Pristimerin	C_30_H_40_O_4_	Triterpenoids	14.67	465.3029	2185.6167	6.3701	Ankli et al., [43])
Rubescensin O	C_21_H_3_2O_7_	Diterpenoids	14.68	397.2244	15742.6836	5.8406	Huang et al., [44]
Costunolide	C_15_H_20_O_2_	Terpene lactones	14.69	233.1540	1415.5000	1.6899	Zdero et al., [45]
Hederagenin	C_30_H_48_O_4_	Triterpenoids	14.69	473.3604	9576.1445	-4.5124	Singh and Sharma, [30]
5,7-dihydroxy-2-(4-methoxyphenyl)-6-(3-methylbut-2-enyl)-2,3-dihydrochromen-4-one	C_21_H_22_O_5_	Flavonoids	14.70	355.1539	1186.2231	-0.2815	Clark et al., [40]
1,2,3,6-tetragalloylglucose	C_34_H_28_O_22_	Tannins	14.86	789.1084	1253.3511	-2.0276	(Hagenah and Gross, [46]
Pechueloic Acid	C_15_H_20_O_3_	Sesquiterpenoids	14.91	249.1483	1633.3057	-0.8870	Miski et al., [31]
Unknown	-	-	11.04	2373.5137	849.3763	-	
Unknown	-	-	3.41	945.3315	994.3118	-	
Unknown	-	-	10.97	2331.2695	1011.4307	-	

## Discussion

Cancer poses a significant threat to human health on a global scale, causing extensive loss of life. In the fight against this insidious disease, the exploration of natural remedies, particularly plant-based therapies carry great importance. The extensive knowledge of traditional medicine highlights the value of plants as powerful sources of therapeutic compounds [47]. In a time where current treatments often result in systemic toxicity and face the challenge of cancer cell resistance, the appeal of natural products as alternative cancer therapies is highly evident. The history of oncology or cancer science has witnessed the remarkable contributions of plant-derived compounds to managing and treating cancer. Several clinically effective anticancer agents, such as paclitaxel, etoposide, teniposide, vinblastine, vincristine, camptothecin, ingenol mebutate, omacetaxine, mepesuccinate and combretastatin A4 phosphate, can be traced back to the world of plants [48, 49]. These compounds, enriched with the wisdom of nature, have revolutionised the field of cancer therapeutics providing renewed hope to those battling this relentless disease [50]. Plant-derived compounds with their diverse chemical structures and mechanisms of action, have the potential to overcome cancer’s strong defences [51]. Their diverse nature allows for innovative approaches in cancer management, ranging from targeting specific cellular pathways to inducing programmed cell death [51]. Additionally, these natural agents often exhibit a lower likelihood of resistance and adverse side effects compared with other treatment options emphasising their significance in overcoming the limitations of current treatment protocols [51, 52].

The current findings (Fig. 1) corroborate data from Magura and colleagues, where they investigated the potential of phytochemical constituents derived from the methanolic extract of *Eriocephalus africanus* (*E. africanus*) to inhibit the growth of cancer cells in vitro [53–55]. *E. africanus* belongs to the same genus as this study species - *E. racemosus* [56]. In the study conducted by Magura, 2020 [55], they successfully isolated a flavanone (hesperidin), as well as two flavones (luteolin and apigenin), from *E. africanus* using column chromatography. An MTT assay was used to evaluate the impact of these isolated phytochemicals on cell viability across multiple cell lines, including MCF-7 (breast cancer), A549 lung cancer, HepG2 liver cancer, and normal HEK 293 cell lines. The flavonoid compounds decreased cell viability dependent on the dose administered across all the tested cell lines [55]. More precisely, hesperidin and luteolin emerged as particularly effective agents in reducing the viability of MCF-7 cells, with half-maximal effective concentration (EC_50_) values of 62.57 µg/mL and 70.34 µg/mL, respectively. On the contrary, apigenin exhibited the most potent activity against HepG2 cells, demonstrating an EC_50_ value of 11.93 µg/mL [55]. The findings of the present study are also consistent with another study [57]. Here, Nthambeleni, 2008 [57] investigated the potential of crude extracts derived from *Eriocephalus tenuifolius* (*E. tenuifolius*, which belongs to the same botanical family and genus as *E. racemosus*) and its isolated compounds against a panel of human cell lines, including renal (TK10), melanoma (UACC62) and breast (MCF 7) cancer cell lines. The in vitro assessment of the organic extract obtained from *E. tenuifolius* demonstrated cytotoxic activity, as evidenced by an average total growth inhibition (TGI) ranging from 12.5 to 15.5 µg/mL across all three cell lines. The organic extract was subjected to bioassay-guided fractionation, eventually isolating and identifying five distinct compounds [57]. Among these compounds, compound 5.12 exhibited an increase in potency, with TGI values of 2.73 µg/mL for the renal cell line, 5.35 µg/ml for melanoma and 4.82 µg/ml for the breast cell line [57]. Compound 5.14 demonstrated a distinctive selectivity profile, exhibiting a specific affinity towards the melanoma cell line with a TGI value of 36.5 µg/mL [57]. Our findings suggest that *E. racemosus* may possess the potential to inhibit or halt the growth of TNBC. Our study contributes significantly to the currently limited and insufficient body of research regarding the cytotoxicity of *E. racemosus* against TNBC cells.

The development of effective cancer treatments relies on the understanding of the selectivity of therapeutic agents towards cancer cells while also sparing healthy cells, which is a crucial factor in designing treatment strategies [58–60]. Cancer, due to its inherent characteristics, arises from uncontrolled cell proliferation and rapid division, emphasising the urgent need for anticancer agents that specifically target malignant cells, thus minimising unintended damage to normal tissues [58, 60]. The current study findings show that, with varying concentrations of 62.5 µg/mL, 125 µg/mL and 250 µg/mL, the growth inhibitory effect observed by *E. racemosus* against Vero cells, a well-established model for normal kidney cells was 17.653%, 19.297%, and 23.538%, respectively (Fig. 1B). These values consistently remained below the critical threshold of 50% [14]. This outcome showed that *E. racemosus* crude extracts exhibited limited cytotoxic effects against Vero cells at the tested concentrations, affirming their selectivity. In comparison, the control drug cisplatin, at a concentration of 3 µg/mL, demonstrated a higher growth inhibition rate of 62.55% against Vero cells (Fig. 1). The findings of the current study align with and support previous research by Adu-Amankwaah et al. (2022) and Sahu et al. (2018) [14, 61] that reported on the cytotoxic effects of *E. racemosus* and *Artemisia nilagrica* (Family: *Asteraceae*), respectively, against Vero cells. In the study done by Adu-Amankwaah et al., 2022, *E. racemosus* leaf crude extracts showed growth inhibitory concentrations of 20–35 µg/mL against Vero cells when screened at a concentration range of 62.5, 125 and 250 µg/mL. Similarly, Sahu et al. (2018) evaluated the cytotoxicity of ethyl acetate fractions extracted from *Artemisia nilagrica* against non-cancerous cells. The bioactive fractions from *Artemisia nilagrica* (Ar-038E and Ar-03E) showed IC_50_ values of 23.22 ± 2.41 µg/mL and 27.83 ± 2.04 µg/mL, respectively, when screened against Vero cells (Sahu et al., 2018) (Fig. 1).

When studying the mode of cell death, apoptosis and necrosis are essential processes in cellular biology, each possessing distinct significance, particularly in the context of cancer treatment. Investigating these cellular phenomena within the context of TNBC, characterised by its aggressive nature and limited therapeutic options, presents a critical avenue of study. Apoptosis is a highly regulated and programmed cell death essential for maintaining tissue homeostasis and eliminating damaged or aberrant cells [7, 62]. It plays a pivotal role in cancer treatment as the induction of apoptosis in cancer cells is a key objective of many therapeutic strategies [7, 62]. Apoptosis enables the controlled removal of cancerous cells without triggering inflammation or harm to neighbouring healthy tissues [7, 63]. In the current study, the administration of cisplatin (3 µg/mL) exhibited a significant increase in both early (20.25 ± 1.43) and late apoptosis (7.29 ± 0.89) within TNBC cells, thereby emphasising its effectiveness in promoting programmed cell death. It is worth noting that *E. racemosus* leaf crude extract and the fraction SF2 showed similar results for both early and late apoptosis when screened against MDA-MB 231 TNBC cells at 12.84 and 15.49 µg/mL, respectively. The crude extract showed an increase of 17.44 ± 0.97, while fraction SF2 was increased of 17.26 ± 0.08% for early apoptosis when compared to the untreated control, which showed 0.05 ± 1.10% and 0.08 ± 0.30, respectively (Figs. 2, 3 and 4). Conversely, necrosis represents a more chaotic and uncontrolled cell death commonly associated with inflammation and tissue damage [64]. While necrosis is generally considered unfavourable in cancer treatment due to its potential to induce inflammation and support tumour progression, its relevance in other specific contexts cannot be disregarded [64]. The noteworthy role of *E. racemosus* fraction and cisplatin in enhancing the incidence of necrosis (8.24 ± 1.12, 3.06 ± 1.76 and 0.89 ± 1.02, respectively) necessitates attention (Figs. 2, 3 and 4). This suggests its potential as an agent capable of inducing programmed cell death while influencing cellular integrity. The findings of the present investigation are consistent with the research conducted by Su et al., 2022 [68]. Their investigation involved two distinct cell lines, CL1-0 (cells associated with human lung cancer) and CL1-0-GR (cells associated with human lung cancer resistant to gemcitabine), representing different experimental conditions [65]. Their findings demonstrate an apoptotic response induced by the extract from *Artemisia argyi* (AAE) (Family: *Asteraceae*), dependent on the dosage, as observed in both cell lineages. Significantly, the group of cells in CL1-0 exposed to 300 and 500 µg/mL of AAE displayed a marked increase in early apoptosis, with a respective rise of 2-fold and 2.8-fold compared to the untreated control group [65]. Moreover, late apoptosis was increased by 2.4-fold and 3.3-fold, specifically in CL1-0 cells exposed to 300 and 500 µg/mL of AAE, when contrasted with the untreated control group [65]. The findings of the present study are also in alignment with a prior study conducted by Adu-Amankwaah et al., 2022 [14]. In our previous report or study, like our current observations, *E. racemosus* (250 µg/mL) demonstrated the capacity to induce apoptosis, with a comparable apoptotic rate of 21.48 ± 2.86%. Notably, the occurrence of necrosis in the [14], study was notably lower, registering at a mere 0.03 ± 0.09%.

Apoptosis, a highly regulated and crucial cellular process, involves the activation of caspase enzymes, with caspases 3, 8 and 9 playing essential roles in coordinating the intricate apoptotic cascade [66]. The activation of caspase 3 functions as a definitive indicator of apoptosis, as it cleaves crucial cellular substrates, ultimately leading to cell demise [66]. Caspase 8 acts as a key initiator caspase in the extrinsic apoptotic pathway, primarily activated by the activation of death receptors [66]. However, caspase 9 emerges as a crucial mediator of the intrinsic apoptotic pathway, actively responding to mitochondrial stress and DNA damage, thereby exerting its regulatory influence [66]. The current study investigated the activity of caspase-3, caspase-8, and caspase-9 in MDA-MB 231 TNBC cells when treated with *E. racemosus* leaf crude extracts, fraction SF2 and cisplatin (Fig. 5A-F). Caspase 3, a critical executor caspase in the apoptotic pathway, exhibited an activity level of 1.26 and 1.04 folds in the presence of *E. racemosus* and SF2, respectively. In comparison, the control group maintained a baseline caspase-3 activity level of 1.00, and cisplatin showed similar activity as the control. This observation emphasises *E. racemosus’s* potential to influence caspase-3 activity, signifying its involvement in apoptosis induction. Increased caspase 8 activity was observed for *E. racemosus* leaf crude extracts, while a decrease was observed for the fraction SF2 (0.92) compared to the control. An increase in both the plant crude extract (0.09-fold) and fraction SF2(0.2-fold), was observed for caspase 9 activity. *E. racemosus* leaf crude extracts increase the activity of all 3 caspases, while the fraction showed a decrease in caspase 8 activity and an increase in caspase 9 activity (Fig. 5). Consistent with our findings, a previous study [67] also observed an increase in caspase activity (caspase 3 and 9) in MCF-7 breast cancer cells treated with the compound, particularly in caspase 3 and caspase 9 when exposed to silver nanoparticles derived from *Achillea biebersteinii*, a plant within the *Asteraceae* family at a concentration of 25 µg/mL. The levels of caspase 3 and caspase 9 activities that were observed (caspase 3: 0.15 and caspase 9: 0.14) were significantly higher compared to those found in the control group. However, it is important to note that they did not observe any changes in the activity of caspase 8 in the same experiment under similar experimental conditions [67].

Glycolysis is a fundamental metabolic pathway that plays a crucial role in cellular energy production and the regulation of various physiological processes. It involves the conversion of glucose into pyruvate, accompanied by the generation of ATP and NADH molecules. Dysregulation of glycolysis has been implicated in various diseases, including cancer, where increased glycolytic flux, known as the Warburg effect, supports the energetic demands of rapidly proliferating tumour cells and contributes to tumour progression and resistance to therapy [19, 68, 69]. The current study showed that treatment with hexane crude leaf extracts of *E. racemosus* induces an increase in basal glycolysis, compensatory glycolysis and 2-DG acidification in MDA-MB 231 TNBC cells at 616.82, 774.64, and 289.42 pmol/min, respectively, in comparison to untreated cells, which exhibited levels of 295.84, 529.80, and 166.18 pmol/min, respectively (Fig. 6). The results presented are in accordance with a study conducted by Reboredo-Rodríguez et al. 2018, in which the impact of ground pistachio kernel extract on MCF-7 breast cancer cell line was explored, focusing on glycolysis and mitochondrial respiration in a dose dependant manner (0.0, 0.5, 1.0, 2.0, and 2.5 mg/mL) [70]. Reboredo-Rodríguez et al. 2018 noted a decline in glycolytic and mitochondrial respiration in MCF-7 cell line after being exposed to ground pistachio kernel extract for 48 h [70].

Mitochondria, organelles found in most eukaryotic cells, play a crucial and indispensable role in cellular energy metabolism. The measurement of mitochondrial membrane potential is a key indicator for assessing the viability of cells. Any loss in membrane potential is closely associated with cellular stress and ultimately leads to cell death. Furthermore, membrane potential dissipation can potentially trigger apoptosis. This phenomenon has been extensively studied and supported by scientific evidence [71]. The generation of membrane potential results from the build-up of a proton-driven electrochemical gradient across the inner mitochondrial membrane. This intricate process depends on the activity of protein complexes within the ETS and the overall integrity of the mitochondrial inner membrane [72, 73]. The ETS is responsible for facilitating the movement of electrons, derived from the oxidation of reduced nicotinamide adenine dinucleotide (NADH) by complex I (NADH-ubiquinone oxidoreductase; C-I), as well as from the oxidation of succinate by complex II (succinate-ubiquinone oxidoreductase; C-II). These electrons, in the presence of molecular oxygen, traverse the ETS until they reach their final electron acceptor. The transfer of electrons through the ETS is intricately linked with the process of proton translocation by the inner membrane complexes I, III (ubiquinol-cytochrome c oxidoreductase; C-III), and IV (cytochrome c oxidase; C-IV). Through this coordinated behind activity an electrochemical proton gradient is established [72, 74]. This energy gradient is then harnessed by the mitochondrial F0/F1-ATP synthase, allowing it to produce ATP [72, 74]. In conjunction with the ATP synthase, the proton pumps within the electron transport system create a proton circuit across the inner membrane, which is central to mitochondrial bioenergetics and cellular homeostasis [73–75]. This interwoven and integrated process of mitochondrial respiration is known as OXPHOS. It is crucial to note that any impairment in the mitochondria’s ability to generate ATP sufficiently can result in energy depletion, leading to cellular stress and potentially triggering various pathways that may ultimately lead to cell death [76, 77]. These pathways have been well-documented in scientific literature and have shed light on the intricate workings of mitochondrial bioenergetics and its repercussions on cellular homeostasis [78].

The present study’s findings highlight the dynamic alterations in cellular respiration and mitochondrial function in MDA-MB-231 TNBC cells following treatment with *E. racemosus* leaf crude extract and cisplatin (Fig. 7; Table 1). These results have significant implications for understanding the impact of these treatments on the metabolic profile of cancer cells, potentially offering valuable insights for therapeutic strategies. Firstly, the substantial increase in routine respiration observed in cisplatin-treated cells signifies a heightened basal metabolic activity, which might be attributed to the cellular response to cisplatin-induced stress. While *E. racemosus*-treated cells also displayed elevated routine respiration, *E. racemosus*-treated cells exhibited a noteworthy deviation from untreated cells. This suggests that both treatments induce alterations in the cells’ baseline metabolic rates [79]. A consistent observation across both treatment groups is the substantial elevation in leak respiration. This shift towards increased leak respiration could indicate heightened mitochondrial permeability, potentially linked to apoptosis or other stress responses [21, 79]. The significance of pyruvate, malate, glutamate, and succinate (after ADP addition) on the glucose oxidation pathway is of utmost importance in this study. *E. racemosus* leaf crude extract exerts a noteworthy influence on these metabolic pathways, thus justifying a thorough exploration of its effects on glucose oxidation. To begin with, it is crucial to acknowledge the importance of the glucose oxidation pathway in cancer cells. The preferential utilisation of glucose, often referred to as the Warburg effect, is a well-documented phenomenon where cancer cells tend to rely on glycolysis for their energy needs, even under aerobic conditions [7, 8]. This metabolic shift is associated with increased lactate production and is thought to support the rapid proliferation and survival of cancer cells. Therefore, any alteration in this pathway could impact cancer cell behaviour significantly. The notable rise in Cytochrome-C levels further highlights potential mitochondria-related events contributing to this phenomenon. The findings about the various functional complexes within the mitochondria are intriguing. Complex-I-linked OXPHOS displayed an increase in both treatment groups, indicating a heightened reliance on this complex for cellular energy production [74, 76]. Additionally, the elevation in electron transfer system capability and the respiration of succinate dehydrogenase in both treatment groups suggest improved overall mitochondrial efficiency, likely compensating for the increased energy demands. Interestingly, Complex-II linked respiration exhibited a significant increase in the treated cells, emphasising a unique impact on mitochondrial function [78]. Moreover, the results involving Glycerophosphate dehydrogenase activity showed substantial increases in both treatment groups, particularly in cisplatin-treated cells, suggesting a metabolic shift influenced by the treatments. The parallel increase in the β-oxidation pathway due to the *E. racemosus* leaf crude extract influence accentuates the complexity and interplay of metabolic pathways within the cancer cells. Conversely, β-oxidative linked OXPHOS displayed only marginal changes, indicating relative independence from the treatments. Notably, Complex-IV activity exhibited an increase in cisplatin-treated cells, while *E. racemosus* leaf crude extract treatment displayed a moderate effect, suggesting distinct responses in mitochondrial respiration between the two treatments. These findings are intriguing, as they contrast with the results observed by Sharma et al. 2021. The differential impact of let-7a on MDA-MB-231 TNBC and MCF-7 cells, as demonstrated by Sharma et al., 2021, highlights the complexity of mitochondrial regulation in different cell types (Sharma et al., 2021). The authors found that malate does not stimulate Complex-I in control cells in MDA-MB-231 TNBC cells, whereas, in the presence of let-7a, significant induction of respiration was observed as represented by reduced oxygen concentration. The addition of ADP and inhibitor of Complex-I rotenone was followed by malate, and the results displayed a slight decrease in oxygen concentration, whereas, in let-7a transfected cells, rotenone stabilises the oxygen concentration, indicating less dependency on Complex-I of MDA-MB-231 [80]. The findings from this study gain significance when considered alongside Kriel et al., 2018 study on U-118MG malignant glioma cells. The study conducted by Kriel et al., 2018 demonstrated that the treatment with 50 µM hydroxychloroquine (HCQ), as compared to control (untreated) cells increased the baseline cellular respiration, which is consistent with the observed elevation in routine respiration found in the current study [21]. These corresponding findings offer compelling evidence that specific therapies can induce metabolic changes, potentially indicating increased energy demands or cellular stress responses. Moreover, both studies observed significant enhancements in the capacity of the ETS and the oxygen flux through Complex I, thereby highlighting a shared theme about the impact of treatment on the efficiency of the mitochondrial electron transport chain and cellular bioenergetics [21, 80].

Liquid chromatography-mass spectrometry (LC-MS) analysis of the crude leaf extracts of *E. racemosus* revealed a wide range of phytoconstituents found in the hexane extract. These phytoconstituents consist of both known and unidentified compounds. Among the compounds tentatively identified in the crude and fraction extracts are Luteolin 7-glucuronide, Moronic acid, Hecogenin, Luteolin 4’-O-glucoside, Pechueloic acid, and Gypsogenin (Fig. 8A-B; Table 2). These compounds have been extensively studied and documented in existing literature, further supporting their relevance to human health [30, 81]. Pechueloic acid was identified in the hexane leave crude extract and fraction SF2 of *E. racemosus*. Sesquiterpenoid and its derivatives have been found to possess antibacterial properties, antimalaria, cytotoxicity and antimycobacterial properties [82]. Further investigation is needed to fully understand the mechanisms by which this compound exerts its anticancer effects. Hecogenin, a steroidal saponin, has also been identified in the hexane leave crude extract. This compound has shown promise in antimicrobial, anti-inflammatory, and antitumour therapy [30]. Its ability to inhibit the growth of microorganisms and cancer cells and reduce inflammation makes it a compound of interest for further research [30]. Hederagenin, a plant triterpenoid in the extract, has attracted attention due to its cholesterol-lowering properties and anti-inflammatory effects [83]. These properties suggest that it may have utility in managing cardiovascular conditions, cancer and inflammatory diseases. Further studies are warranted to explore the full potential of this compound in the field of medicine. Glochidone, another compound identified in the extracts, is known for its cytotoxicity against cancer cells and antioxidant and anticholinesterase activities [81, 84]. This finding suggests that it may have a potential role in cancer therapy. Further investigation is needed to determine the mechanisms by which this compound exerts its cytotoxic effects and to evaluate its efficacy in treating cancer [84]. Gypsogenin, a triterpenoid compound present in the fraction extract, has been found to possess anti-inflammatory and wound-healing properties [85]. This makes it a promising candidate for the treatment of various skin-related conditions and inflammatory disorders. Its ability to reduce inflammation and promote wound healing has been well-documented in scientific literature [85].

Moreover, the LC-MS analysis of the extracts revealed unidentified compounds, introducing a novel aspect to the research and emphasising the need for further investigation to clarify their chemical structures and potential bioactivities. Understanding the identities and characteristics of these unknown compounds could offer valuable insights into the overall composition and potential therapeutic applications of *E. racemosus*. While individually identified compounds present health advantages, their presence in the crude extracts of *E. racemosus* hexane leaves suggests potential synergistic effects, highlighting the therapeutic potential of this plant. This investigation constitutes the initial report on the phytoconstituents in the crude extracts of *E. racemosus*, providing a fundamental understanding of the plant’s chemical composition and shedding light on previously unidentified bioactive compounds. This innovative exploration opens possibilities for studying the therapeutic and pharmacological potential of *E. racemosus*, emphasising the importance of further inquiries into its diverse phytochemical profile (Fig. 8; Table 2).

## Conclusion

*Eriocephalus racemosus* demonstrates promising cytotoxic activity against TNBC, inducing apoptosis through multiple pathways. The plant induces a notable dual enhancement in glycolysis and mitochondrial respiration in MDA MB 231 TNBC cells, suggesting potential interference with the cancer cell’s energy production. The metabolite profiling results provide valuable insights into the bioactive compounds present in *E. racemosus*. These findings support further exploration of *E. racemosus* as a potential therapeutic agent for TNBC, offering a promising avenue for the development of targeted treatments with minimal adverse effects.

### Recommendations

This study presents an exciting avenue for exploring *E. racemosus* as a potential treatment for TNBC. However, it is essential to recognise that further research is required to validate its safety and efficacy, particularly in vivo, preclinical and clinical settings. The findings of this study set the stage for a promising journey toward developing innovative treatments for TNBC. Also, the crude extract obtained from the plant encompasses a wide range of compounds, which can subsequently be divided and subjected to experimental evaluation for their anticancer effects. Even though the results suggest that the crude leaf extracts possess the capability to combat TNBC, further investigation is required into the mechanisms of action of the plant and isolate the bioactive constituents accountable for the plants’ anticancer characteristics in the in vivo examination.

## Electronic supplementary material

Below is the link to the electronic supplementary material.

Supplementary Material 1

## Data Availability

The datasets used and analyses during the current study are available from the corresponding authors upon reasonable request.

## Abbreviations

MTT: 3-(4,5-Dimethylthiazol-2-yl)-2,5-diphenyltetrazolium bromide
ATP: Adenosine triphosphate
DMEM: Dulbecco’s modified Eagle’s medium
DMEM: Dulbecco’s modified Eagle’s medium
*E. racemosus*: *Eriocephalus racemosus*
FBS: Fetal bovine serum
GPNR: Grootbos Private Nature Reserve
LR: Leak Respiration
L/P: Leak/OXPHOS
P/E ratio: Phosphorylation system control ratio
RO: Routine Respiration
TNBC: Triple-negative breast cancer
