# Supplementary material for: Cytotoxic properties, glycolytic effects and high-resolution respirometry mitochondrial activities of Eriocephalus racemosus against MDA-MB 231 triple-negative breast cancer
Source: BMC Complement Med Ther. 2024 Sep 10;24:332. doi: 10.1186/s12906-024-04615-x (PMC11389270; doi:10.1186/s12906-024-04615-x)
Supplement: Supplementary file 1 — Supplementary Material 1 [file 12906_2024_4615_MOESM1_ESM.docx]

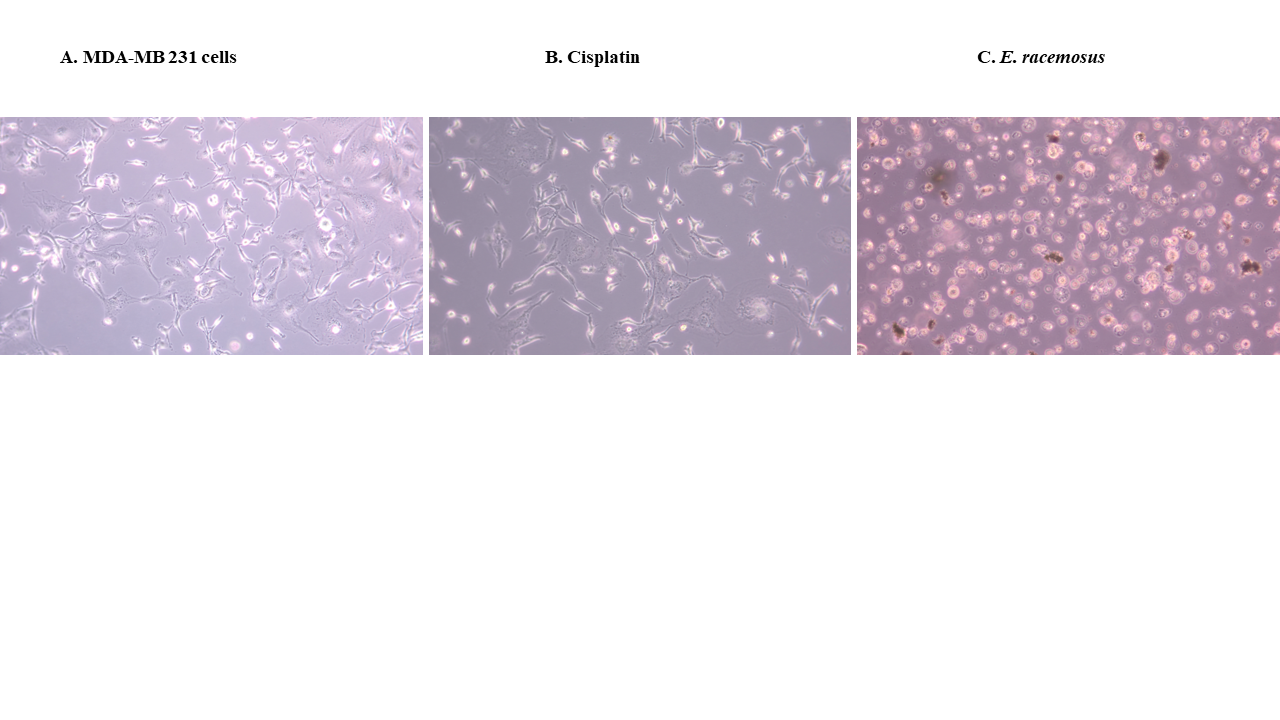


**Figure 1 A-C.** Effect of *E. racemosus* and cisplatin on the cell morphology of MDA-MB 231 cells. Cells were treated with cisplatin and the crude extract of *E. racemosus* at a concentration of 3 µg/mL and 250 µg/mL for 24 hours. The results were observed under a light inverted microscope (Zeiss, Germany); magnification, 10x.

**Table 1.** Summary of IC_50_ values of the cytotoxic effect of cisplatin and *E. racemosus* crude leave extract against MDA-MB 231 triple negative cancer cell line

| **Samples** | **IC_50_ Values (µg/mL)** | **p-value** |
| --- | --- | --- |
| Cisplatin | 2.106 ±0.09 |  |
| *E. racemosus* | 12.84 ±0.314 | 0.0001 |

**Table 2.** List of bioactive fractions

| **Solvents** | **Yield** | **Extracts** |
| --- | --- | --- |
| 100% Methanol | 3 % | SF1 |
| 80% Hexane + 20% ethyl acetate | 45% | SF2 |
| 60% Hexane + 40% ethyl acetate | 30% | SF3 |
| 40% Hexane + 60% ethyl acetate | 5% | SF4 |
| 20% Hexane + 80% ethyl acetate | 5% | SF4 |
| 100% Ethyl acetate | 7% | SF6 |

**Table 3.** Summary of IC_50_ values of the cytotoxic effect of cisplatin and *E. racemosus* fraction against MDA-MB 231 triple negative cancer cell line

| **Samples** | **IC_50_ Values (µg/mL)** | **p-value** |
| --- | --- | --- |
| Cisplatin | 2.106 ±0.09 |  |
| SF2 | 15.49 ±0.950 | 0.0013 |
